# Translation of human Δ133p53 mRNA and its targeting by antisense oligonucleotides complementary to the 5′-terminal region of this mRNA

**DOI:** 10.1371/journal.pone.0256938

**Published:** 2021-09-07

**Authors:** Paulina Żydowicz-Machtel, Mariola Dutkiewicz, Agata Swiatkowska, Dorota Gurda-Woźna, Jerzy Ciesiołka

**Affiliations:** Institute of Bioorganic Chemistry, Polish Academy of Sciences, Poznan, Poland; Universidad de Jaen, SPAIN

## Abstract

The p53 protein is expressed as at least twelve protein isoforms. Within intron 4 of the human *TP53* gene, a P2 transcription initiation site is located and this transcript encodes two p53 isoforms: Δ133p53 and Δ160p53. Here, the secondary structure of the 5′-terminal region of P2-initiated mRNA was characterized by means of the SHAPE and Pb^2+^-induced cleavage methods and for the first time, a secondary structure model of this region was proposed. Surprisingly, only Δ133p53 isoform was synthetized *in vitro* from the P2-initiated p53 mRNA while translation from both initiation codons occurred after the transfection of vector-encoded model mRNA to HCT116 cells. Interestingly, translation performed in the presence of the cap analogue suggested that the cap-independent process contributes to the translation of P2-initiated p53 mRNA. Subsequently, several antisense oligonucleotides targeting the 5′-terminal region of P2-initiated p53 mRNA were designed. The selected oligomers were applied in *in vitro* translation assays as well as in cell lines and their impact on the Δ133p53 synthesis and on cell viability was investigated. The results show that these oligomers are attractive tools in the modulation of the translation of P2-initiated p53 mRNA through attacking the 5′ terminus of the transcript. Since cell proliferation is also reduced by antisense oligomers that lower the level of Δ133p53, this demonstrates an involvement of this isoform in tumorigenesis.

## Introduction

The p53 protein is one of the major factors responsible for cell cycle regulation and stress response. The protein is expressed as at least twelve protein isoforms, resulting from the usage of alternative promoters, downstream initiation codons or alternative splicing [1–3]. Within intron 4 of the human *TP53* gene, a P2 transcription initiation site is located. This transcript encodes two p53 isoforms: Δ133p53 and Δ160p53. In addition to the Δ133p53 isoform, also referred to as Δ133p53α, isoforms truncated from the carboxyl terminus, Δ133p53β and Δ133p53γ, have also been defined. Analogously to Δ133p53 isoforms, Δ160p53α, Δ160p53β, and presumably also Δ160p53γ variants are generated in the cell [4,5].

It has been shown that Δ133p53 mRNA variants (α, β and γ) are expressed in a wide range of normal tissues but in a tissue-dependent manner, suggesting that their expression can be regulated [6]. Consequently, the Δ133p53 proteins are expressed in a variety of normal and cancer cells and they can regulate many important cellular processes [1–3]. Numerous recent publications have suggested that Δ133p53 protein may be implicated in tumorigenesis processes, including angiogenesis and metastasis, as well as in other cellular events, e.g. in proliferation, cellular senescence or apoptosis. Interestingly, most studies have shown that the oncogenic function of Δ133p53 is mediated through its antagonistic effect against wild-type p53. Since Δ133p53 and Δ160p53 isoforms have a truncated DNA-binding domain it is unclear whether they are capable of binding to the specific DNA sequences. However, due to the intact oligomerization domain they can potentially form tetramers with themselves and also with full length p53, inhibiting the regular p53 pathway [1]. Our current knowledge about the biological activities and physiological functions of p53 isoforms, including those leading to cellular senescence, ageing and cancer, has recently been reviewed [1–3].

Similarly, studies conducted on selected model organisms reveal that the Δ133p53 isoforms can regulate many important cellular processes. It has been shown that Δ113p53, the orthologue of Δ133p53 identified in zebrafish, can inhibit apoptosis induced by p53 and this isoform is active in the modulation of target gene expression of the p53 protein [7]. These observations are consistent with those concerning Δ122p53, the experimentally generated mouse isoform [8]. An increased synthesis of Δ122p53 in mouse cells results in increased proliferation and decreased apoptosis of the cells. Animals who have been identified as having a high level of Δ122p53 are characterized by an increased predisposition to various types of cancer and high mortality [8]. Recent review papers discuss the contribution of *Drosophila* studies [9] and those on the mouse model [10] to the knowledge on p53 expression and p53 novel roles in promoting tissue homeostasis, as well as cell invasion and metastasis.

The last few years have brought a large number of data indicating the very important role of Δ133p53 and Δ160p53 isoforms in cell functioning. However, information about mRNA transcripts encoding these isoforms is far from being sufficient. In particular, this applies to the 5′-terminal, non-coding regions of these transcripts. Our previous studies of mRNA variants initiated from the P1 and P0 sites of the *TP53* gene, encoding the full-length p53 and Δ40p53 isoform, have pointed to a very important role of the 5′-terminal mRNA regions in the expression of these isoforms under normal and stress conditions [11–14]. Thus, characterization of the 5′-terminal region of P2-initiated p53 mRNA seems to be crucial to understanding the role of this region in translation and translational regulation. Undoubtedly, it is also important to fully uncover the role of Δ133p53 and Δ160p53 isoforms in the functioning of the cell.

In order to modulate protein expression, antisense oligomers or interfering RNAs (siRNAs) are routinely used. The siRNAs seem to be more effective in inhibiting expression, yet antisense oligomers are still considered as more appealing molecular tools in biomedical applications [15–18]. The 5′-terminal regions of mRNAs are attractive places for targeting with oligonucleotide tools, because these regions influence translation initiation, the most highly regulated phase of translation. In the so-called scanning model [19], the initiation complex begins scanning from the 5′ untranslated regions of mRNAs and these regions together with interacting protein factors may greatly modulate translation efficiency [20–24]. Recently, we have shown that translation initiation of P1-initiated p53 mRNA variants is highly modulated by the length and structure of their 5′-terminal regions and that translation efficiency may be effectively regulated by antisense oligonucleotides [12,13,25].

Here, we characterized the secondary structure of the 5′-terminal region of human p53 mRNA that starts from the P2 transcription initiation site and which encodes Δ133p53 and Δ160p53 isoforms. The variability of the nucleotide sequence of this region in other organisms was also compared. The efficiency of translation initiation from the sites corresponding to Δ133p53 and Δ160p53 isoforms was determined under *in vitro* and *in cellulo* conditions. Subsequently, we described the rational design of antisense oligomers targeting the 5′-terminal region of P2-initiated p53 mRNA aimed to inhibit translation initiation. Translation efficiency in the presence of selected oligomers was determined *in vitro* and in selected cell lines. Cell viability upon transfection of the studied antisense oligonucleotides was also examined.

## Materials and methods

### dsDNA, primers and antisense oligonucleotides

The dsDNA plasmid pcDNA3.3-TOPO with a Δ133p53 sequence was purchased from Invitrogen. The insert sequence was designed based on the reference Δ133p53 sequence (NCBI Reference Sequence: NM_001126115.1), with T7 polymerase promoter and a coding sequence for FLAG peptide. The antisense DNA oligonucleotides and PCR primers were purchased from Genomed S.A. The modified antisense RNA oligonucleotides No.: 3, 4, 18, and C were ordered in (Thermo Fisher Scientific).

### Site directed mutagenesis

The dsDNA templates for the constructs Δ133p53 AUG3/CUA4 and Δ160p53 CUA3/AUG4 were obtained by site-directed mutagenesis of the Δ133p53 dsDNA construct. The following forward (F) and reverse (R) primers were used:

FmutAUG133p53: 5′-CCTGCCCTCAACAAGCTGTTTTGCCAACTGGCC-3′,

RmutAUG133p53: 5′-GGCCAGTTGGCAAAACAGCTTGTTGAGGGCAGG-3′,

FmutAUG160p53: 5′-GCGTCCGCGCCCTGGCCATCTACAAGCAG-3′,

RmutAUG160p53: 5′-CTGCTTGTAGATGGCCAGGGCGCGGACGC-3′.

The PCR reaction mix was prepared in accordance with the manufacturer’s protocol using *Pfu* DNA Polymerase (Promega), 500 ng of the dsDNA template and 75 μM forward or reverse primer. Reactions with forward primer and reverse primer were performed separately, but under the same conditions for 10 cycles of initially 2 min of denaturation at 95°C, then 50 s at 95°C, 50 s at 60°C, 16 min at 68°C, and finally 7 min at 68°C. Afterwards, two reactions were combined, 1.5 U of *Pfu* polymerase was added, and PCR reaction was performed in the same conditions as above, for 20 cycles. Subsequently, the reaction products were purified by GeneMATRIX PCR/DNA Clean-Up Purification Kit (Eurx). Then, 1000 ng of each dsDNA sample was treated with 50 U of Dpn I restriction enzyme (Thermo Fisher Scientific), according to the manufacturer’s protocol. After digestion, dsDNA was transformed into *E*. *coli* TOP-10 competent cells (Thermo Fisher Scientific). The sequence of the construct was confirmed by sequencing.

### *In vitro* transcription

Prior to transcription, dsDNA templates were linearized with EcoRI restriction enzyme (Thermo Fisher Scientific), in accordance with the manufacturer’s protocol. Subsequently, 500 ng of DNA was taken to the *in vitro* transcription using TranscriptAid T7 High Yield Transcription Kit (Thermo Fisher Scientific) supplemented with 3 mM Anti Reverse Cap Analog (ARCA, New England Biolabs). After the transcription reactions, the RNA probes were treated with 1 U of DNase I enzyme for 20 min at 37°C and purified using GeneJET RNA Purification Kit (Thermo Fisher Scientific).

### Pb^2+^-induced RNA cleavage

Prior to the Pb^2+^-induced RNA cleavage, 70 pmol of RNA was denatured in a buffer containing 40 mM NaCl, 10 mM Tris-HCl pH 7.2, 10 mM MgCl_2_ for 5 min at 65°C and then renatured for 5 min at 37°C. Afterwards, the lead acetate solution was added to each sample to the final concentration of 0.5 mM, 1 mM, 2 mM, and an equal volume of water was added to the control reaction. The reaction was conducted for 3 min at 37°C and terminated by an addition of 10 mM EDTA. The RNA was precipitated with 300 mM sodium acetate pH 5.2, 0.2 mg glycogen (Thermo Fisher Scientific) and 180 μl of ethanol. The RNA pellet was dissolved in water and used in primer extension reaction. For reverse transcription reaction, RNA sample and 2 pmol of each [^32^P]-labelled DNA primers:

R133p53UTR2: 5′-AATCAACCCACAGCTGCACAGGGCAG-3′,

R133p53UTR3: 5′-GTGAACAGATAAAGCAACTGG-3′,

R133p53UTR6: 5′-TTTGAGATAGGGTCTTGCTCTGTCAC-3′,

R160p53UTR: 5′-CAACCTCCGTCATGTGCTGTGAC-3′,

R133p53UTR2_2: 5′-ACAGGGCAGGTCTTGGC-3′.

were first denatured for 5 min at 65°C. The reaction mixture contained: 1X FS buffer, 200 U SuperScript IV Reverse Transcriptase (Invitrogen), 0.5 mM dNTPs Mix, and 10 U RiboLockRNase inhibitor (Thermo Fisher Scientific). The reaction was conducted for 10 min at 55°C followed by 15 min at 70°C. The sequencing reaction was prepared in the same conditions as described above, using RNA samples and 0.2 mM dideoxy-terminating nucleotides. Finally, the solution containing 8 M urea, 20 mM EDTA and xylene cyanol dye was added to the samples, incubated for 3 min at 95°C and loaded on 8 M urea, 8% polyacrylamide gels. After electrophoresis the gels were scanned by phosphorimaging using the FLA 5100 image analyzer (FujiFilm).

### SHAPE analysis

0.4 μM of RNA was denatured in a buffer containing 10 mM Tris pH 8.0, 100 mM KCl, and 0.1 mM EDTA, at 90°C for 3 min and cooled (0.1°C/s) to 4°C. Next, buffer: 40 mM Tris pH 8.0, 5 mM MgCl_2_, 130 mM KCl, and 0.1 mM EDTA was added to sample and incubated at 37°C for 10 min. In this buffer a modification reaction was performed using NMIA dissolved in DMSO (5.5 mM final concentration). The control reaction contained DMSO without NMIA. Both modification and control reactions were incubated at 37°C for 50 min and the RNA was precipitated and resuspended in water. An analysis of the modification sites was performed by the primer extension reaction, in the same manner as described above for Pb^2+^-induced RNA cleavage.

For the purpose of SHAPE data analysis [26], following the electrophoresis each band was scanned with a radioactivity scanner. Bands that corresponded to NMIA-modified nucleotides and those present in the control lane were integrated using MultiGauge software to obtain a numerical output of the band intensities. The generated numerical data, i.e. raw intensities, were normalized. In the simple normalization scheme, the most reactive 2% of all intensities are removed from the pool. The intensities of the next 8% most reactive peaks are averaged and all reactivities are divided by this average value. A normalized reactivity of 1.0 is defined as the average intensity of the top 10% most reactive peaks, excluding a few highly reactive nucleotides taken to be outliers. It is proportionally calculated in such a manner that a majority of the values are included in the range between 0 and 1, where 0 stands for the lowest reactivity, meaning that there are no modifications and the nucleotides are paired, while 1 means lack of nucleotide pairing. Subsequently, normalized SHAPE intensities were entered as pseudo-free energy constraints to obtain secondary structures for the studied RNA molecules. The secondary structure predictions were performed using the *RNAstructure* 5.6 program.

### *In vitro* translation

*In vitro* translation was performed using Rabbit Reticulocyte Lysate (RRL) System (Promega). The reaction contained 8.75 μl RRL, 20 μM aminoacid mixture minus methionine, 0.5 μl ^35^S-methionine (1000 Ci/mol, Hartman Analytic), 10 U of RiboLockRNase inhibitor (Thermo Fisher Scientific) and 1.25 pmol of capped RNA, which was previously denatured for 5 min at 65°C. The final volume of the reaction was 12.5 μl and it was conducted for 90 min at 30°C. Afterwards, the reaction was treated with 0.16 μg of RNase A for 5 min at 20°C, and denatured for 2 min at 80°C in the presence of SDS Sample Buffer and 100 mM DTT. The reaction products were analyzed in 15% SDS-PAGE, followed by radioisotope imaging with FLA 5100 image analyzer (FujiFilm).

For translation inhibition assay, RRL was pre-incubated with an increasing concentration of m^7^GpppG cap analog from 5 to 750 μM (Epicenter Biotechnologies) and the same concentrations of magnesium acetate, for 15 min at 30°C. Afterwards, 20 μM amino acid mixture minus methionine, 0.5 μl ^35^S-methionine (1000 Ci/mol, Hartman Analytic), 10 U of RiboLockRNase inhibitor (Thermo Fisher Scientific) and 1.25 pmol of previously denatured RNA were added to the RRL samples and incubated for 90 min at 30°C. The samples were treated with RNase A and analyzed in 15% SDS-PAGE as described above.

### RNase H assay

Prior to digestion with *E*. *coli* RNase H (Thermo Fisher Scientific), 1 pmol of RNA was denatured in a buffer containing: 40 mM Tris-HCl pH 8.0, 40 mM KCl, 10 mM MgCl_2_, 1 mM DTT, 0.1 mM EDTA for 5 min at 70°C and then for 5 min at 37°C. Then, 5 pmol of each antisense oligonucleotide was added and incubated for 5 min at 37°C. The cleavage reactions were induced by adding 0.5 U of RNase H enzyme. The reaction mixtures were incubated for 7.5 min at 37°C. The reactions were stopped by adding an equal volume of 20 mM EDTA. The reaction products were analyzed by 1% agarose gel in 1xTBE.

### Cell lines and transfection

MCF-7, HepG2 and H1299 cells were purchased from ECACC. MCF-7 cells were maintained in DMEM, HepG2 in MEM and H1299 cells were cultured in McCoy’s medium. All media (Sigma) were supplemented with 10% fetal bovine serum, non-essential amino acids (Gibco-BRL), 100 U/ml of penicillin G, 0.1 mg/ml of streptomycin sulphate (Sigma). The plasmid transfection in the final concentration of 1 ng/μl was performed using Lipofectamine 3000 (Thermo Fisher Scientific) or Dharmafect 2 (Dharmacon) transfection reagents. After 4 hours 2′-*O*Me PS antisense oligonucleotides at a final concentration between 0.075 and 0.5 μM were transfected into cells using Lipofectamine RNAiMax reagent, according to the manufacturer’s transfection protocol (Invitrogen) [27]. Cells were harvested after 24 hours.

### Western blots

For Western blots, cell lysates were prepared in a buffer: 62.5 mM Tris-HCl pH 6.8, 2% SDS, 10% glycerol, 50 mM DTT and protease inhibitor (Roche). Total cell lysates were incubated for 5 min at 95°C and then loaded on a 15% SDS-PAGE gel and proteins were transferred to a PVDF membrane (GE Healthcare). The blot was probed with mouse DYKDDDDK Tag Polyclonal Antibody (FLAG) (Thermo Fisher Scientific), rabbit anti-p53 monoclonal antibody Pab421 (Abcam), rabbit β-Actin Monoclonal Antibody (Cell Signaling), mouse GAPDH Monoclonal Antibody (Santa Cruz Biotechnology). Primary antibody was detected by Goat Anti-Mouse-HRP or Goat Anti-Rabbit-HRP (Thermo Fischer Scientific) and visualized using the enhanced chemiluminescent visualization (ECL) system (Thermo Fisher Scientific Pierce).

### Flow cytometry

Flow cytometry measurements were performed after 24, 48 and 72 hours of oligomer transfection using a LIVE/DEAD Viability/Cytotoxicity two-color flow cytometry assay (Thermo Fisher Scientific), following the manufacturer’s protocols. The procedure was optimized in the Laboratory of Subcellular Structures Analyses of our Institute [28]. Briefly, the cells were detached with trypsin (Thermo Fisher Scientific), washed twice with Dulbecco’s Phosphate Buffered Saline (Thermo Fisher Scientific), and stained for 30 min at 37°C in the dark. The cells were analyzed immediately after staining with excitation at 488 nm by FACSCalibur™ flow cytometer (Becton Dickinson).

## Results

### Secondary structure of the 5′-terminal region of P2-initiated p53 mRNA

Initially, it has been thought that the promoter of P2-initiated mRNA, found in the *TP53* gene, covers the region from intron 1 to the proximal part of exon 5, including about 1500 base pairs [6]. Further studies have shown, however, that the promoter area comprises only 250 base pairs of the 3′ terminus of intron 4 [29]. The resulting P2-initiated mRNA transcripts contain the 3′ end of intron 4 and all following exon sequences. These transcripts have been identified in both healthy and tumor cells, but not in every cell type. It has been observed that the presence of P2-initiated mRNAs depends on the type of cell line [4,6].

**Fig 1:**
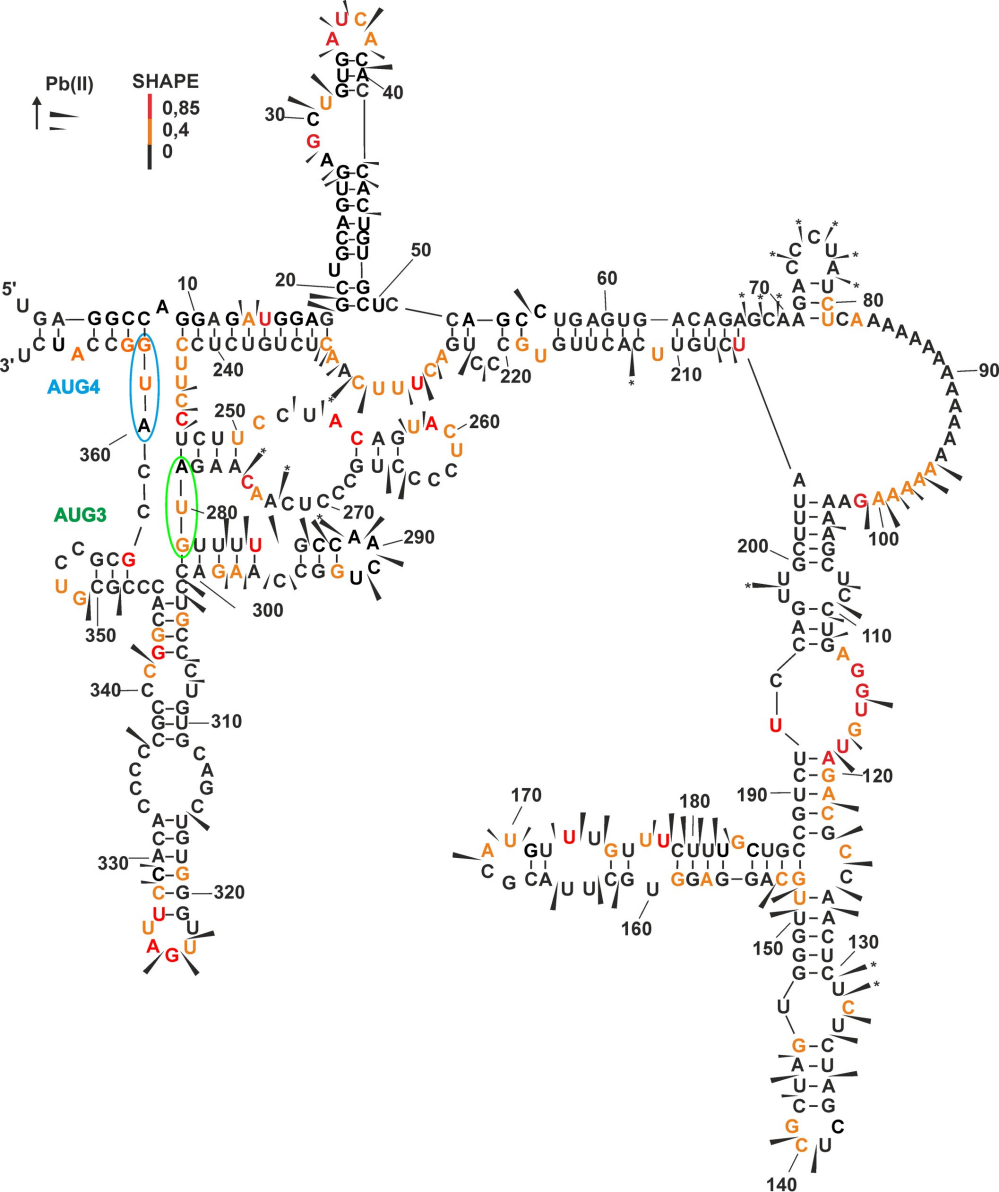
**The secondary structure model of 5′-terminal region of Δ133p53 mRNA analyzed by Pb**^**2+**^**-induced cleavage and SHAPE.** A secondary structure model corresponding to experimental data was generated by *RNAstructure 5*.*6* software. Nucleotide symbols are marked with colors according to their SHAPE reactivity. Cleavages induced by Pb^2+^ ions are displayed on the secondary structure model with arrows. Arrows with an asterisk * indicate RT-stops. The green loop in the model denotes AUG3 initiation codon for Δ133p53 isoform and the blue loop marks AUG4 for Δ160p53 isoform.

The model P2-initiated p53 mRNA transcript (Δ133p53 mRNA) was synthesized by *in vitro* transcription. The transcript starts in intron 4 of the *TP53* gene and includes 250 final nucleotides of the intron 4 sequence. In Δ133p53 mRNA, the AUG3 initiation codon for Δ133p53 protein is located at nucleotide positions 279–281, while AUG4 codon for Δ160p53 isoform is located at positions 360–362 (Fig 1). The structure of the 5′-terminal region of Δ133p53 mRNA was probed by the SHAPE [30] and Pb^2+^-induced cleavage [31,32] methods (Supporting information, S1A and S1B Fig and ST1 Table in S1 File). The results of the SHAPE method were applied into *RNAstructure 5*.*6* program [33] and a few probable secondary structures of the analyzed region were generated (Supporting information, S1C Fig in S1 File). Structures in the range of 10% of minimum free energy were taken into account, out of which the one most compatible with the SHAPE and Pb^2+^-induced cleavage data was chosen.

Fig 1 shows the proposed secondary structure model of the 5′-terminal region of Δ133p53 mRNA with the stabilization energy ΔG of -140 kcal/mol. In the structure, the 5′-terminus is extensively base-paired with the 3′ part of the analyzed region. This pairing results in formation of a large domain which is attached through a single-stranded tract of eighteen adenosine residues, A83-A100, to a second domain that spans nucleotides A103 and U204. Both domains are on the opposite sides of the secondary structure model and in the first domain, the AUG3 and AUG4 translation initiation codons are embedded. The AUG3 codon is positioned between two hairpin motifs while the AUG4 codon is located mostly in a single-stranded region (Fig 1).

The proposed secondary structure model is consistent with the experimental probing data. Most single-stranded stretches are revealed by strong SHAPE modifications and Pb^2+^-induced cleavages (Fig 1 and Supporting information, lS1 Fig in S1 File). In particular, intense SHAPE signals are present in the A35-A38 region, while in the case of mapping with Pb^2+^ ions, strong cleavages in the G27-C44 region occur. Both methods nicely map the internal and apical loops of hairpin G19-C49. Moreover, SHAPE modifications and Pb^2+^ induced cleavages occur between A96 and G101, in the single-stranded, A-rich sequence stretch. Efficient modifications and cleavages also occur in the A126-U151 and G152-C187 regions, which create unstable hairpin motifs with several unpaired nucleotides. Two single-stranded regions A225-A232 and U243-C246 are well mapped with the SHAPE method. Whereas the unstable nature of hairpin G281-C301, which is composed of short segments with weak base-pairs, is revealed by several Pb^2+^-induced cleavages. Finally, the presence of single-stranded region U322-U326, which corresponds to the apical loop of hairpin U316-A332, is confirmed by both SHAPE and Pb^2+^-induced cleavage approaches (Fig 1 and Supporting information, S1 Fig in S1 File).

**Fig 2:**
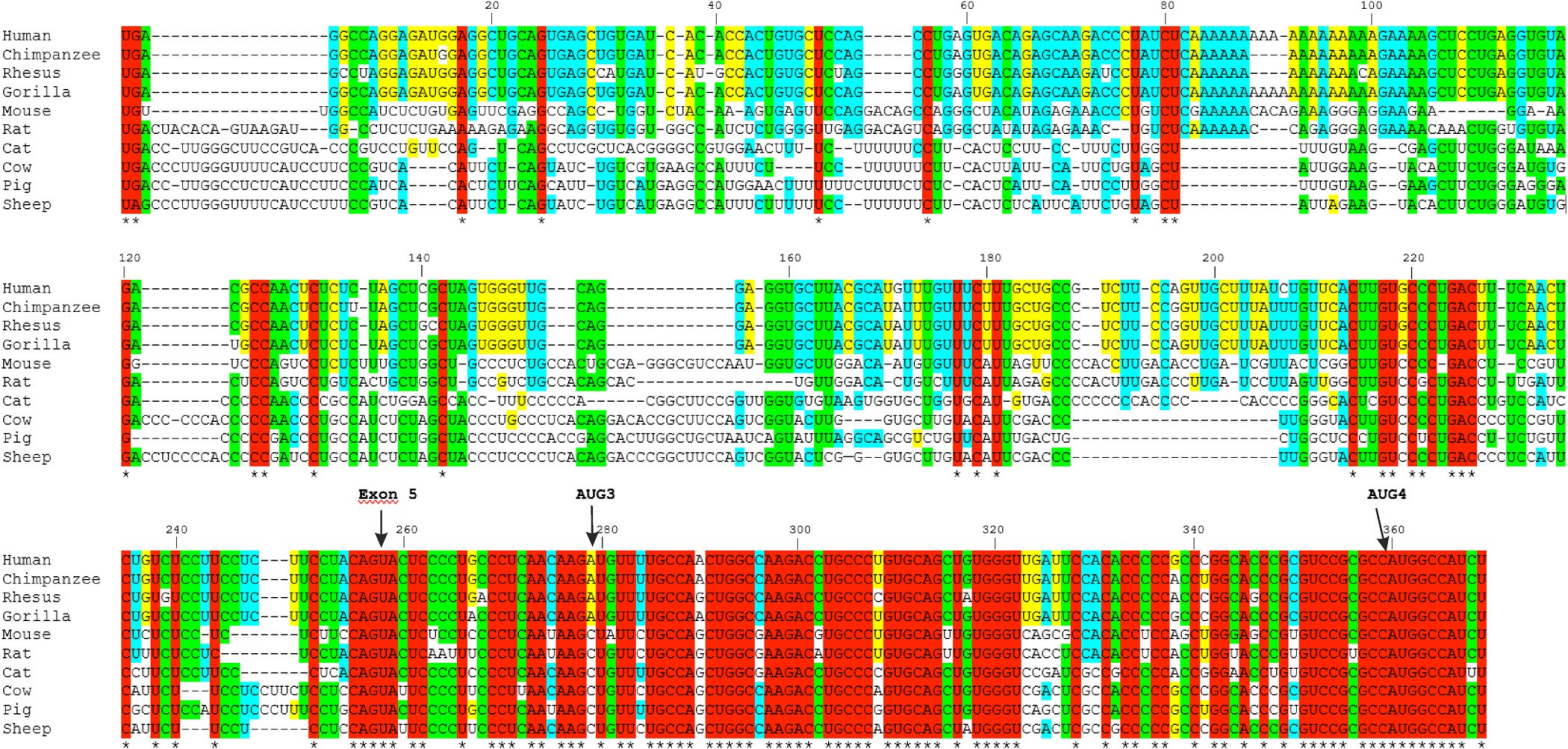
Sequence conservation of the 5′-terminal region of Δ133p53 mRNA. Comparison of the 5′-terminal regions of Δ133p53 mRNA sequences derived from ten different species. The gene sequences were obtained from Ensembl Website. Translation initiation codons AUG3 and AUG4 as well as the exon 5 site are indicated by arrows. The alignment was performed using Clustal Omega program. Alignment is colored according to the percentage of sequence conservation (red: 100%; green: 80%– 99%; blue: 60%– 79%; yellow: 59% - 40%; black: 39%-0).

We compared the secondary structure model of the 5′-terminal region of P2-initiated p53 mRNA with the conservation of nucleotide sequences of that region in various organisms. For that purpose, the alignment of 5′-terminal sequences of p53 mRNAs from 10 mammals was generated (Fig 2). The idea of this comparison was to find regions or structural elements which are highly conserved in mammals. Such regions may play important functions, likely also *via* interacting with some cellular proteins. The protein coding sequence between AUG3 and AUG4 is most conserved. This region extends to the intron/exon junction at dinucleotide GU, and comprises eight nucleotides upstream in the sequence which are, however, less conserved. Particularly interesting structural elements found in the secondary structure of the corresponding region include a hairpin of low stability which precedes AUG3 and three hairpin motifs, which are positioned immediately downstream in the sequence. These hairpins are arranged into a cloverleaf-like structure (Fig 1). A few single nucleotides present in this region are less conserved. High variability of the 4-nucleotide stretch maps nicely the apical loop of the central hairpin of the cloverleaf-like structure. On the other hand, in the intron-derived sequence, four stretches of relatively high conservation can be distinguished: A104-A121, C124-A144, C214-C226 and U238-C246 (Fig 2). In the secondary structure model, nucleotides of the last two stretches are important for base pairing of the 5′ terminus with the 3′ part of the studied region. And the two former stretches are located in the large structural domain that spans the previously mentioned interacting regions. Other stretches of the intron-derived sequence are much less conserved. It is worthy of note that the entire intron-derived sequences are highly conserved in humans, chimpanzee, rhesus and gorilla (Fig 2).

### Efficiency of translation from AUG3 and AUG4 start codons

**Fig 3:**
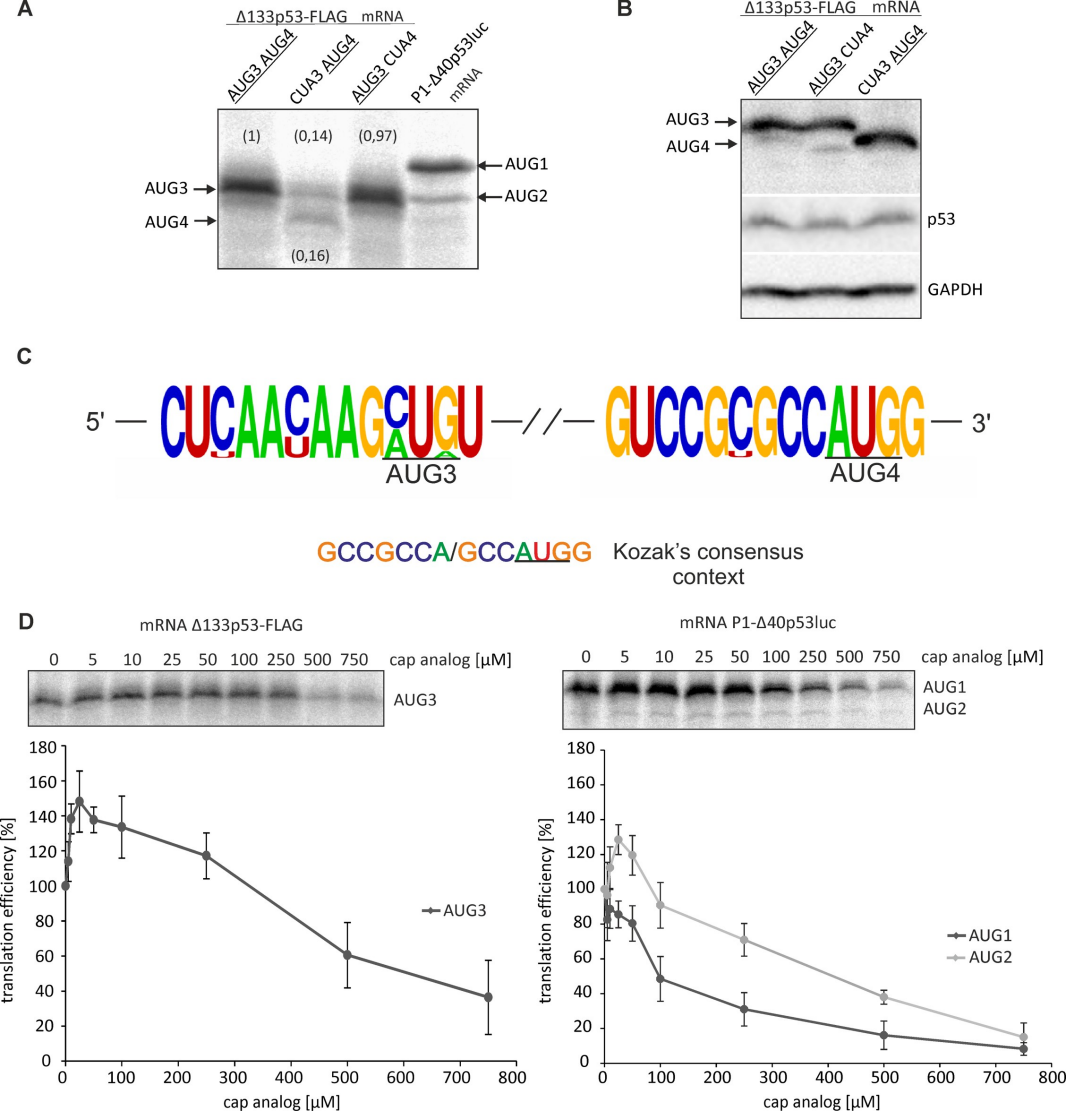
Translation of mRNA constructs from AUG3 and AUG4 initiation codons under *in vitro* and in cellular conditions. A) Autoradiogram presents translation products of model Δ133p53-FLAG mRNAs with AUG3 and AUG4 initiation codons or with those codons mutated to triplets CUA3 and CUA4, when translation was performed in rabbit reticulocyte lysate (RRL) with [^35^S]-methionine. The P1-Δ40p53luc mRNA construct with AUG1 and AUG2 codons was used in a control reaction. Above and below the bands the relative translation efficiency from AUG3 and AUG4 codons is shown. B) Translation from AUG3 and AUG4 in HCT116 cells transfected with vector-encoded model Δ133p53-FLAG mRNAs. Western blot shows Δ133p53-FLAG, p53 and GAPDH levels, C) The Kozak’s context of AUG3 and AUG4 codons in the 5′-terminal regions of Δ133p53 mRNA sequences derived from ten different species shown in Fig 2D) The capped mRNA variants were translated in RRL in the presence of an increasing concentration of the cap analogue (m^7^GpppG) to inhibit cap-dependent translation. The amounts of protein products resulting from AUG3 or AUG1 and AUG2 initiation codons were determined, and following quantification and normalization to the values with no cap analogue added, they were displayed on the graph. The graph presents the mean of three independent measurements, with the standard deviations calculated and displayed on the diagrams.

Due to the presence of two initiation codons in the Δ133p53 mRNA, two isoforms, Δ133p53 and Δ160p53, can be synthesized from this transcript [4]. In order to determine the relative efficiency of translation from both these codons we performed translation of *in vitro* synthetized Δ133p53-FLAG mRNA in rabbit reticulocyte lysate (RRL) with radiolabeled methionine (Fig 3A). Surprisingly, after translation of this mRNA in RRL only one band was detected on the gel, despite the presence of two potential initiation codons, AUG3 and AUG4. As expected, two protein products were clearly visible in the control reaction, in which the model P1-initiated p53 mRNA with AUG1 and AUG2 initiation codons was used, P1-Δ40p53luc (Fig 3A). In order to determine which initiation codon of Δ133p53 mRNA, AUG3 or AUG4, was active in translation *in vitro*, site directed mutagenesis of both these codons was performed. Two derivatives of Δ133p53 mRNA were synthetized, mRNA(CUA3AUG4) and mRNA(AUG3CUA4), in which the first or the second AUG codon was changed to a CUA triplet coding leucine. Unexpectedly, translation of mRNA(CUA3AUG4) in RRL yielded two protein products of comparable intensity (Fig 3A). The faster migrating product was presumably the Δ160p53 isoform synthesized from AUG4. The band intensity was however approx. 6-fold lower than that for Δ133p53 isoform synthesized from AUG3 with mRNA(AUG3CUA4). The second product most likely reflected translation initiation from a non-standard initiation codon [34,35]. Thus, even with the mRNA bearing only one initiation codon AUG4, translation *in vitro* from this codon was so ineffective that another non-standard initiation codon took over.

In order to demonstrate translation initiation from both AUG3 and AUG4 codons in cellular environment HCT116 cells were transfected with a plasmid vector expressing Δ133p53-FLAG mRNA (Fig 3B). After incubation for 24 hours western blot analysis of protein products was performed with the use of anti-FLAG antibody. To confirm that the detected protein was Δ133p53-FLAG synthesized from the expression vector we performed western blot analysis of the H1299 cell line, in which p53 is not expressed, transfected with the vector. For this analysis, we also used the Pab421 antibody, which specifically recognizes Δ133p53 and other p53 isoforms (Supporting information, S2 Fig in S1 File). For transfected HCT116 cells two protein products, likely originated from both AUG3 and AUG4 codons were observed on the gel (Fig 3B). The assignment of these products was confirmed using two derivatives of Δ133p53-FLAG mRNA, mRNA(CUA3AUG4) and mRNA(AUG3CUA4). In the case of mRNA(AUG3CUA4) besides the expected translation product initiated from AUG3 an additional faint band was observed on the gel which likely reflected translation initiation from a non-standard initiation codon.

It has to be noted that the translation of model P2-initiated Δ133p53 mRNA clearly differs from the translation of P1-initiated p53 mRNA under *in vitro* and *in cellulo* conditions [11,12]. In the former case, both the full-length p53 protein and Δ40p53 isoform synthesized from AUG1 and AUG2 codons are always observed in the ratio of approx. 5:1 (Fig 3A). Strikingly, the translation efficiency of Δ133p53 mRNA from AUG3 and AUG4 does not follow the Kozak’s rule on the preferred nucleotide context of translation initiation codons [19]. The context for AUG3 determined based on the alignment of the 5′-terminal sequences of p53 mRNAs from 10 mammals (Fig 2) differs substantially from the preferred Kozak’s consensus context (Fig 3C; see also the Discussion section). On the other hand, AUG4 is embedded in the nucleotide context, which is almost perfectly conserved and which much better corresponds to the optimal context for efficient translation initiation.

In order to get some information on translation initiation mechanisms which may operate for the synthesis of Δ133p53 and Δ160p53 isoforms we performed a translation assay of Δ133p53-FLAG mRNA in RRL in the presence of an increasing concentration of the cap analog, m^7^GpppG (Fig 3D, left panel). At low 25 μM concentration of the cap analog, an increase in protein synthesis from AUG3 initiation codon was observed and it reached the maximum of 140%, remaining at this level up to 100 μM concentration of the analog, compared with synthesis efficacy with no inhibitor added. At a higher concentration of the cap analog, synthesis efficacy gradually decreased. However, at 500 μM concentration of the analog, translation efficiency was still approximately 60% of that with no inhibitor added. As a reference, the same experiment was performed with P1-initiated p53 mRNA construct with AUG1 and AUG2 codons (Fig 3D, right panel). The inhibition curves resembled those obtained earlier in our laboratory with model mRNAs bearing the 5’-terminal region of P1-initiated p53 mRNA, showing strong cap-dependent characteristics for AUG1 and essential contribution of cap-independent translation initiation for AUG2 [11,12]. Importantly, the curve course corresponding to translation from AUG2 resembles that of translation from AUG3 when Δ133p53-FLAG mRNA was translated in RRL, suggesting a contribution of the cap-independent process to translation initiation. However, further experiments are needed to verify this assumption.

### Antisense oligonucleotides that hybridize to the Δ133p53 mRNA inhibit translation *in vitro*

**Fig 4:**
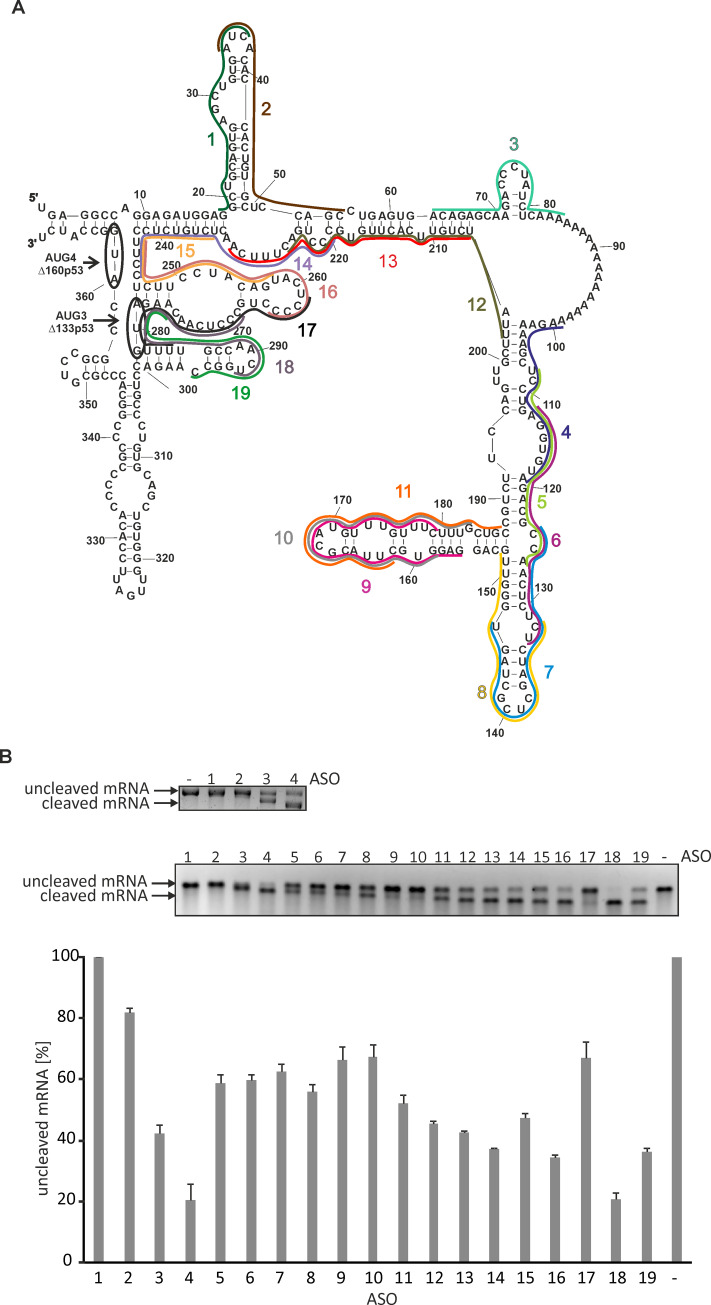
Antisense oligonucleotides targeting the 5′-terminal region of Δ133p53 mRNA. A) Secondary structure model of the 5′-terminal region of Δ133p53 mRNA with several DNA antisense oligomers targeting this region. Oligomers No. 1 to 19 are represented by different color lines along the sequence. B) RNase H assay with antisense oligomers No. 1–19. The gels show the products of RNase H cleavage upon each oligomer binding to the RNA. In the upper gel with oligomers No. 1–4 the cleavage products are resolved better after longer electrophoresis. The reaction with no oligomer added was used as a control. The cleaved and uncleaved RNA reaction products are indicated by arrows. The graph shows the average values of RNase H cleavage extents obtained from three independent experiments.

Based on the secondary structure model of the 5′-terminal region of Δ133p53 mRNA, several antisense DNA oligomers hybridizing to this region were designed (Fig 4A and Supporting information, ST2 Table in S1 File). Oligomers were designed using the OligoWalk function in *RNAStructure 5*.*6* program. While designing the oligomers, the general rules that govern hybridization of antisense oligonucleotides to RNA targets were taken into account [36,37]. The duplex ΔG values reflecting oligomer-target binding energy were also calculated and all oligomers were designed to exhibit similar ΔG values (from -25.4 to -28.6 kcal/mole) and a similar length (from 20 to 26 nucleotides). Moreover, the oligomers were targeted at presumably important structural elements of the 5′-terminal region of P2-initiated p53 mRNA.

In order to assess the binding affinity of the oligomers to the 5′-terminal region of Δ133p53-FLAG mRNA, the RNase H assay was performed (Fig 4B). It turned out that almost all the oligomers hybridized to the RNA target since they induced the RNase H activity which cleaved the RNA:DNA duplexes. Oligomers No. 4 and 18 induced cleavage of almost 80% of the initial amount of RNA, therefore they seemed to hybridize with the best affinity. Nearly 60% RNA was cleaved in the presence of oligomers No. 3, 14, 16 and 19, indicating strong binding of these oligonucleotides to the target. The RNA cleavage efficacy was around 50% or below upon the binding of the other oligomers. Therefore, for further studies, the following oligonucleotides were selected: No. 3 hybridizing to a small unstable hairpin motif, No. 4 targeting the internal loop region within a large structural domain, and No. 18 hybridizing to the region with the AUG3 initiation codon (Fig 4A).

**Fig 5:**
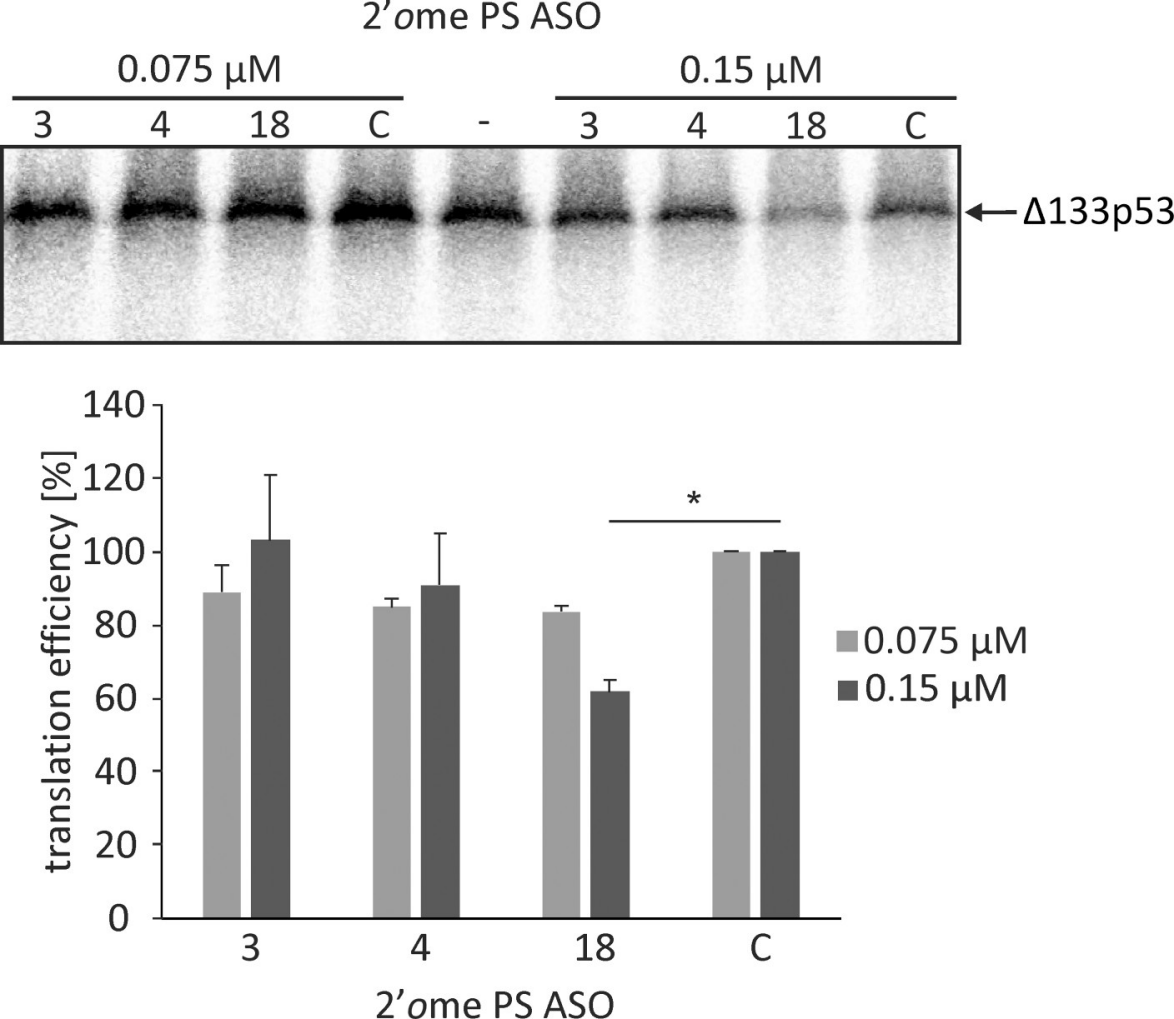
*In vitro* translation of the Δ133p53-FLAG mRNA in the presence of selected antisense oligonucleotides. Autoradiogram shows the products of *in vitro* translation of Δ133p53-FLAG mRNA in RRL in the presence of selected 2′*O*Me PS RNA oligomers No.: 3, 4 and 18 used at concentration of 0.075 and 0.15 μM; Symbol (C) represents the control oligomer and (-) a reaction with no oligomer added. The bar chart quantitatively displays the translation efficiency from AUG3 initiation codon in the presence of each oligomer. All values are means of at least three independent experiments and they were normalized to the values with control oligomer. The *p* value was calculated by the Student’s t-test and the significant result is marked on the chart with an asterisk (*p* < 0.05).

The three selected antisense oligonucleotides were applied in *in vitro* translation assays with Δ133p53-FLAG mRNA in RRL system (Fig 5). The oligonucleotides were synthesized as their 2′*O-*methylated derivatives with phosphorothioate internucleotide bonds to avoid inducing RNase H activity and to increase their resistance to nuclease degradation. Additionally, an oligomer that does not hybridize to the 5′-terminal region of Δ133p53 mRNA (oligo C) was used as the control. Out of the three oligomers tested, the lowest protein level was observed in the presence of oligomer No. 18, which was bound to the mRNA with the highest affinity among the tested oligonucleotides. An approximately 40% decrease of translation efficiency at 0.15 μM concentration of oligomer No. 18 was observed in comparison to the reaction with the control oligomer while at the 0.075 μM concentration of oligomer No. 18, a 20% decrease of translation occurred. Oligonucleotides No. 3 and 4 also caused reduction of the Δ133p53 protein synthesis, yet to a smaller extent compared with oligomer No. 18 (Fig 5).

### Antisense oligonucleotides reduce the synthesis of exogenous Δ133p53 in cell lines

**Fig 6:**
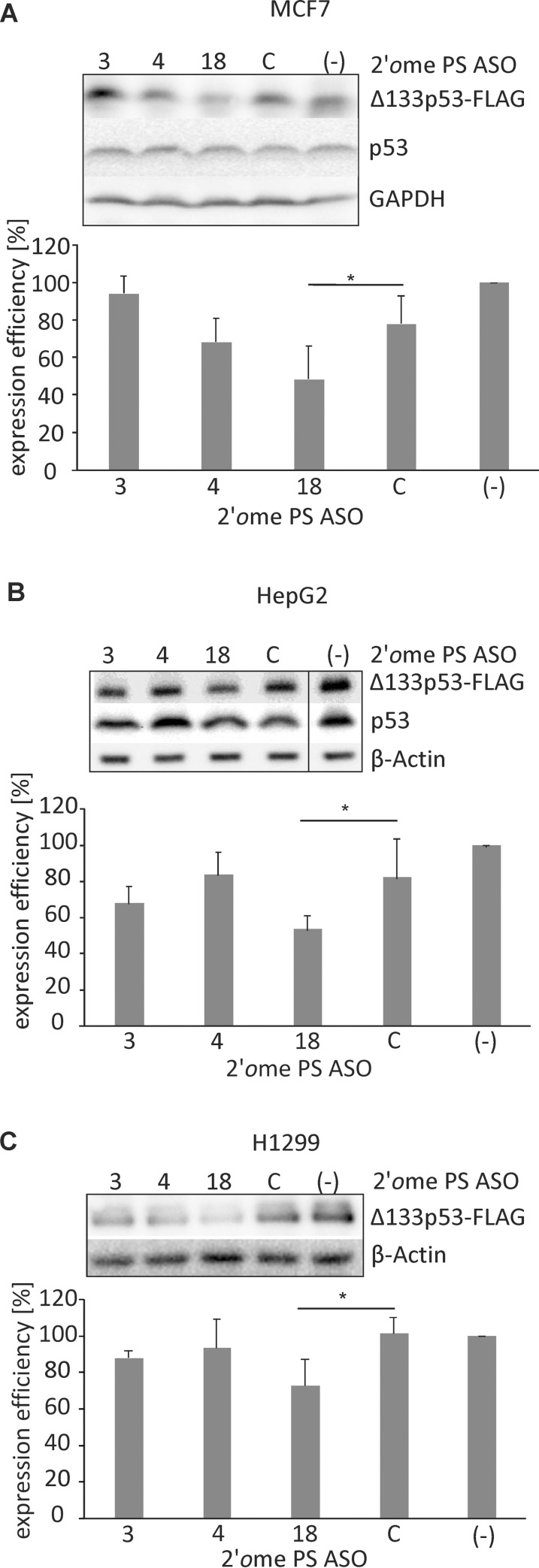
Expression of the Δ133p53-FLAG isoform in the presence of 2*′O*Me PS RNA antisense oligonucleotides in MCF-7, HepG2 and H1299 cell lines. A) MCF-7 cells were transfected with Δ133p53 expression vector with FLAG-tag and with 0.15 μM oligomers No.: 3, 4, 18 and control oligomer C. Western blot shows Δ133p53-FLAG, p53 and GAPDH levels. All values were normalized to those with no oligomers added (-). The GAPDH level was used as a loading control. The graph shows the average values of three independent experiments. The *p* value was calculated by the Student’s t-test and marked on the chart with an asterisk (*p* < 0.05). B) Western blot shows Δ133p53-FLAG, p53 and β-actin levels in HepG2 cells in the presence of 0.075 μM oligomers No.: 3, 4, 18 and control oligomer C. The β-actin was used as a loading control. C) Western blot shows exogenous Δ133p53-FLAG and β-actin levels in H1299 cells in the presence of 0.15 μM oligomers No.: 3, 4, 18 and control oligomer C.

Modified antisense oligonucleotides were applied to test whether they are able to reduce the Δ133p53 isoform synthesis in MCF-7 and HepG2 cells (Fig 6A and 6B). The cells were transfected with plasmid expressing Δ133p53 with FLAG tag and after a four hour incubation the antisense oligonucleotides were transfected to the cells. Following incubation for additional 24 hours, the protein level was analyzed by western blot with the use of anti-FLAG antibody while the Δ133p53-FLAG mRNA level was determined by real-time PCR. In order to confirm that the detected protein was Δ133p53-FLAG synthesized from the expression vector we performed western blot analysis of H1299 cell line transfected with the vector. For this analysis, we also used the Pab421 antibody, which specifically recognizes Δ133p53 and other p53 isoforms (Supporting information, S2 Fig in S1 File). Importantly, small variations in Δ133p53-FLAG mRNA levels in H1299, HepG2 and MFC*-*7 cell lines, which were determined by real-time PCR, did not correspond to the changes in Δ133p53-FLAG protein level occurring in the presence of antisense oligonucleotides (Supporting information, S4 Fig in S1 File).

We observed an almost 30% reduction of Δ133p53-FLAG after application of oligomer No. 18 at 0.15 μM concentration to MCF-7 cells compared to those cells with the control oligomer added (Fig 6A). Interestingly, even a two-fold lower concentration (0.075 μM) of the same oligomer caused an approx. 25% decrease of Δ133p53-FLAG in HepG2 cells (Fig 6B). This level of inhibition is similar to that obtained in *in vitro* translation assays. In both cell lines oligomer No. 4 caused an insignificant decrease of the Δ133p53 synthesis by 5 to 10%. Oligomer No. 3 reduced the protein level by 15% in HepG2 cells, while no effect was observed in MCF-7 cells despite the fact that a higher concentration of oligomer No. 3 was used in this cell line. It is worthy of note that the control oligomer changed the Δ133p53 synthesis by 20% in both HepG2 and MCF-7 cell lines, compared to those cells with no oligomer added, which can be explained by off-target effects. Importantly, western blot analysis of the p53 protein in these cell lines showed that the level of p53 does not change upon transfection of the tested antisense oligonucleotides to the cells.

In order to eliminate the potential contribution of endogenous Δ133p53 protein to silencing results with the use of cells transfected with the plasmid encoding Δ133p53-FLAG we decided to use a p53-null H1299 cell line (Fig 6C). Almost 30% reduction of protein synthesis was observed after addition of oligomer No. 18 at 0.15 μM concentration to these cells in comparison to the reaction with the control oligomer added. At the same 0.15 μM concentration, oligomers No. 3 and 4 decreased slightly the Δ133p53 level to 85% and 90% of the control, respectively.

**Fig 7:**
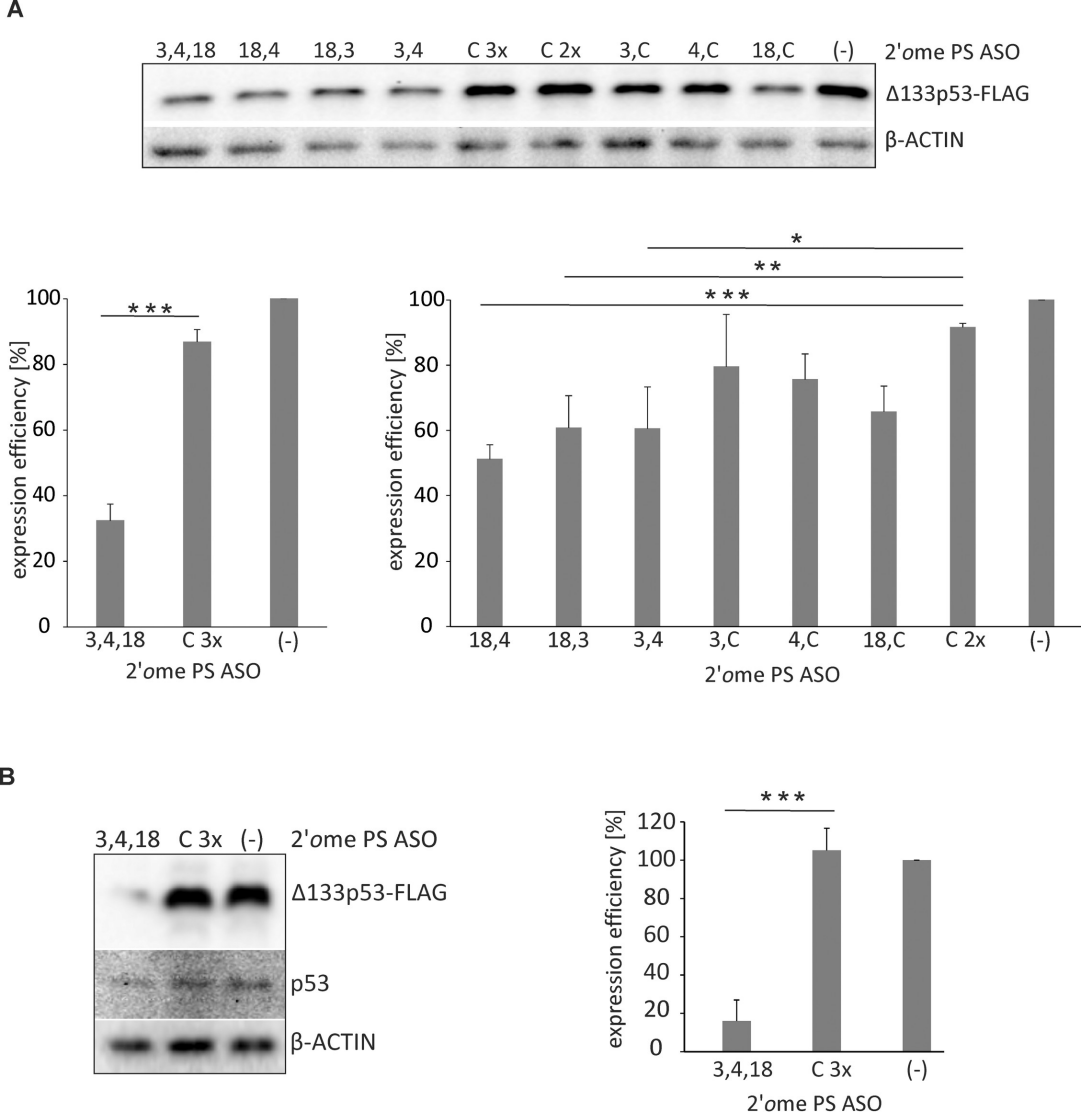
Western blot analysis of Δ133p53-FLAG expression in H1299 and MCF-7 cells transfected with 2*′O*Me PS RNA antisense oligonucleotide mixes. The blots present Δ133p53-FLAG isoform levels in H1299 (A) and in MCF-7 (B) cells in the presence of the mixes of oligomers No. 3, 4, 18 and control C oligomer. The cells were transfected with Δ133p53 expression vector with FLAG-tag and subsequently oligomers No. 3, 4, 18 and oligomer C were transfected in mixes, each at a concentration of 0.15 μM and the final concentration of oligomer mixes in the reaction did not exceed 0.45 μM. Mixes used in the experiment are listed on the graphs. Control oligomer C was used at a concentration of 0.30 μM and 0.45 μM; lines C2x and C3x, respectively. The experiments were performed in at least three independent repetitions and the values were normalized to those without oligonucleotides added (-). The β-actin level was used as a loading control. The *p* value was calculated by the Student’s t-test and significant comparisons are marked with asterisks: (*p* < 0.05)*, (*p* < 0.01)**, (*p* < 0.001)***.

In order to further reduce the Δ133p53-FLAG level in H1299 cells transfected with the Δ133p53-FLAG-encoding plasmid and to observe possible synergetic effects, various mixes of the tested and control oligomers were used (Fig 7A). The largest inhibitory effect was observed after transfection of the cells with a mixture of three oligomers: No. 3, 4 and 18, each at the 0.15 μM concentration. The protein expression decreased by 55% compared to the cells with the control oligomer used at a 3-fold higher concentration (0.45 μM). On the other hand, only approx. 10% lower protein level was observed with the control oligomer compared to the cells with no oligomers added. Compared to the cells with the control oligomer used at a two-fold higher concentration (0.30 μM), the Δ133p53-FLAG level was reduced by 40% in the presence of mixed oligomers No. 4 and 18, and by 30% in the presence of mixed oligomers No. 3 and 18 or No. 3 and 4. When oligomers No. 3, 4 or 18 were used in combination with the control oligomer, the Δ133p53-FLAG amount decreased by 10, 15 and 25%, respectively (Fig 7). This is consistent with the data in Fig 6C: the same protein levels were obtained after cells transfection with oligomers No. 3, 4 and 18 separately and an insignificant side effect was observed caused by the control oligomer. A mixture of three oligomers: No. 3, 4 and 18, each at the 0.15 μM concentration reduced the Δ133p53-FLAG level also in MCF-7 cells transfected with the Δ133p53-FLAG-encoding plasmid (Fig 7B). The protein expression decreased by over 80% compared to the cells with the control oligomer used at a 3-fold higher concentration.

### Viability of MCF-7 cells upon transfection of antisense oligonucleotides targeting the 5′-terminal region of Δ133p53 mRNA

**Fig 8:**
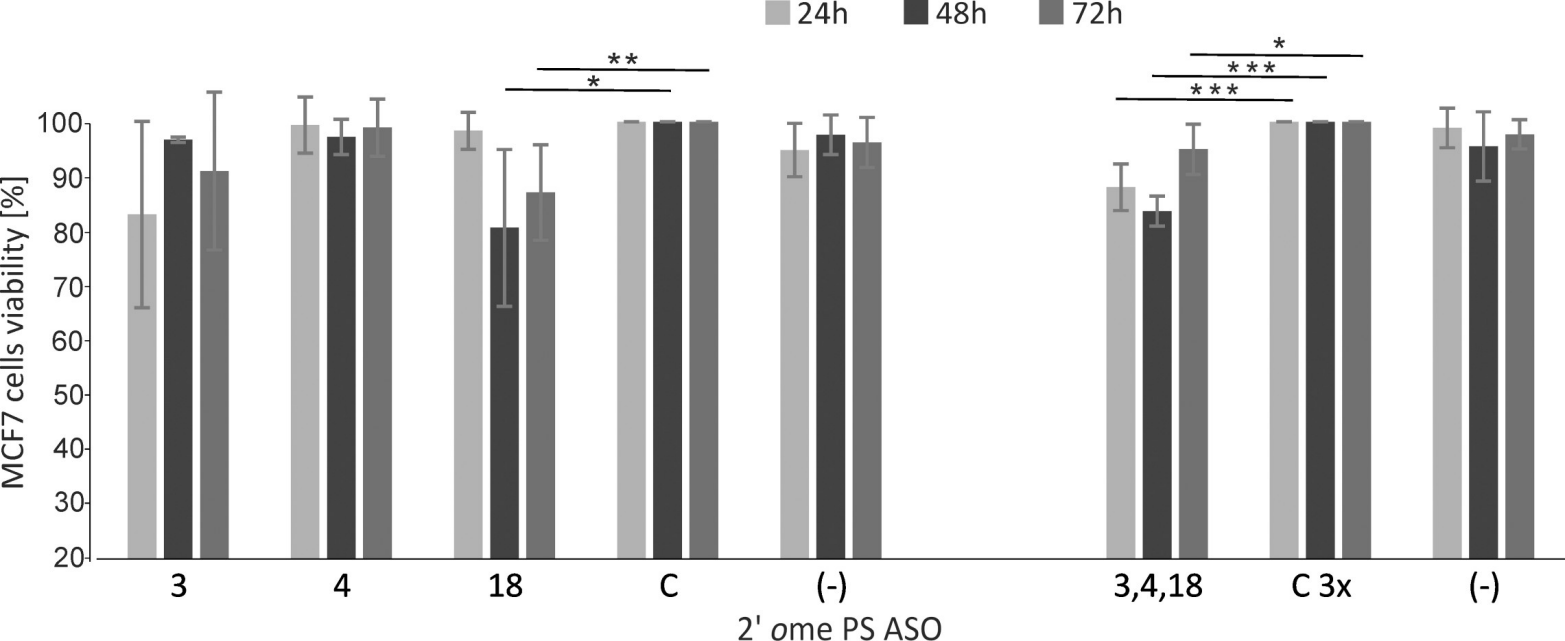
MCF7 cell lines viability assay upon transfection of 2′*O*Me PS RNA antisense oligonucleotides. Flow cytometry analysis of MCF7 viability by dual staining with Calcein/Ethidium Bromide. Cells were transfected with specified antisense oligomers and the mix of oligomers 3, 4 and 18. Control oligomer C was also used at a concentration of 3-fold higher than each of the other specific oligonucleotides: line C3x. The LIVE/DEAD assay was performed 24, 48 and 72 hours post transfection. Samples C(-) indicate no oligonucleotide added. The graph shows average values obtained from three independent experiments, normalized to oligomer C or C3x. The *p* value was calculated by the Student’s t-test and selected comparisons are marked with asterisks: (*p* < 0.1)*, (*p* < 0.05)**, (*p* < 0.01)***.

In order to examine the impact of Δ133p53 on cell proliferation, we downregulated the expression of this isoform in carcinoma cell line, MCF-7. We analyzed cell viability after 24, 48 and 72h post transfection with all the antisense oligonucleotides and their mixes, by flow cytometry using LIVE/DEAD dual staining (Thermo Fisher Scientific). Analysis of fluorescence intensity indicated the number of live and dead cells for each experimental condition. Surprisingly, for these fast growing cells we observed changes in cell proliferation [28] already after 48 hours from transfection (Fig 8 and Supporting information, S3 Fig in S1 File). Oligomer No. 18 caused a decrease in cell survival by approximately 20% whereas oligomers No. 3 and 4 affected the cell viability only by approximately 3%. After 72 hours, oligomer No. 18 still caused a decrease in cell viability by approximately 13%. When applied as a mixture, oligomers 3, 4, and 18 caused the decrease by app. 16% after 48 hours, however this effect was also visible earlier, after 24 hours (12%) and was remaining after 72 hours by 5%. Currently, we continue these studies attempting to explain in detail the correlation between the effects of antisense oligonucleotides on Δ133p53 synthesis and on cell viability using cancerous and non-cancerous cell lines.

## Discussion

Here, we proposed for the first time the secondary structure of the 5′-terminal region of p53 mRNA that started from the P2 transcription initiation site (Δ133p53 mRNA) (Fig 1). The 5′ terminus of this region is extensively base-paired with its 3′ part. The first translation initiation codon AUG3 is located in an unusual structural environment, just between two hairpin motifs. Moreover, the second hairpin is a part of a three-hairpin domain which separates the AUG3 and AUG4 codons. High thermodynamic stability of this domain of ΔG = -29 kcal/mol and conservation of nucleotides present in this mRNA region (Fig 2) suggest that the three-hairpin domain may play an important functional role. Surprisingly, translation of Δ133p53-FLAG mRNA in rabbit reticulocyte lysate showed only one protein product that was initiated from AUG3 (Fig 3A). Translation of the mRNA derivatives with mutated initiation codons confirmed that AUG3 is strongly preferred in translation *in vitro* or it is an exclusive initiation site, whereas translation from AUG4 proceeded with low efficiency only when AUG3 codon was mutated. However, when HCT116 cells were transfected with Δ133p53-FLAG mRNA two protein products, originated from both AUG3 and AUG4 codons were observed although translation from AUG4 occurred with much lower efficiency (Fig 3B).

It is worthy of note that translation of Δ133p53-FLAG mRNA from AUG3 and AUG4 codons clearly differs from that which has earlier been observed for P1-initiated p53 mRNA [11,12]. The full-length p53 and Δ40p53 isoforms synthesized from AUG1 and AUG2 codons are always observed under *in vitro* and *in cellulo* conditions in the ratio of approx. 5:1 [11,12]. Translation of Δ133p53-FLAG mRNA *in vitro* occurring only from AUG3 and much weakly from AUG4 than from AUG3 in HCT116 cells transfected with vector-encoded Δ133p53-FLAG mRNA contradicts the expectations based on the Kozak’s rule on the preferred nucleotide context of translation initiation codons [19]. The context for AUG3: CUCAACAAGAUGU differs substantially from the Kozak’s consensus context of GCCGCC(A/G)CCAUGG and AUG4 is embedded in the nucleotide context of GUCCGCGCCAUGG, which much better corresponds to that optimal for efficient translation initiation (Fig 3C). This is opposite to the nucleotide contexts of the initiation codons in P1-initiated p53 mRNA [11]. The AUG1 codon is embedded in the context: GUCACUGCCAUGG, which better corresponds to the Kozak’s consensus than the context: UCCCAAGCAAUGG for AUG2 and, as expected, AUG1 is preferred in translation. Thus, in Δ133p53 mRNA, factors other than the nucleotide composition of the initiation codons surroundings must have a big impact on translation initiation. Possibly, the structural features of the mRNA close to and/or between AUG3 and AUG4 affect the translation mechanism. Supposedly, the three-hairpin domain which separates both codons may contribute to the translation mechanism, enhancing initiation from AUG3 and/or inhibiting it from AUG4.

The Δ160p53 isoform, the second protein product which is synthesized from Δ133p53 mRNA [4] has been detected in several cell lines endogenously expressing different mutant p53s, but to our knowledge this isoform has not been observed in translation *in vitro* conditions. Moreover, WT p53 cell lines (HCT116, U2OS and A549) show either no signs or low levels of Δ160p53 expression [5]. Also, HFKs (human foreskin keratinocytes) and HPECs (human prostate epithelial cells) do not show any detectable Δ160p53α expression [38]. Thus, our observation that no Δ160p53 isoform is synthetized in the RRL system and that its expression is much weaker than that of Δ133p53 in transfected HCT116 cells supports the postulated demanding requirements for expression of this isoform. Besides the structural features of the mRNA close to and/or between AUG3 and AUG4, this may involve interactions of Δ133p53 mRNA with specific protein cofactors.

So far, not all functions of the Δ133p53 and Δ160p53 isoforms have been defined and regulation of their expression is still uncovered. As elevated Δ133p53 is only observed in cells with WT *TP53* it seems likely that it is the activation of p53 that trans-activates the Δ133p53 isoform [4,39]. It is unclear whether Δ133p53 may promote cell growth independently of p53, although a p53-independent pro-survival function of Δ133p53 has recently been demonstrated that depends on ΔNp63 [40]. A metabolic switch toward glycolysis is induced that drives cell proliferation and tumorigenesis. It has also been shown that increased Δ133p53 elevates the levels of interleukin 6 and other pro-inflammatory cytokines. A model has been proposed showing how Δ133p53 regulates the JAK-STAT3 and RhoA-ROCK networks. This results in the activation of the NFkB pathway and the generation of multiple pro-inflammatory chemokines that contribute to the migration of tumor cells as well as to promotion of an invasive phenotype [41]. Interestingly, the introduction of WT Δ133p53β in the poorly invasive WT *TP53* MCF-7 breast cancer cells that express all WT p53 isoforms except Δ133p53β, enhances the invasive activity of MCF-7 [42]. A strong association between Δ133p53β mRNA levels in glioblastoma with an increased tumor-associated macrophage content has also been observed. Elevated Δ133p53β is an alternative pathway to *TP53* mutation in glioblastoma that aids tumor progression by promoting an immunosuppressive and chemo-resistant environment [43]. Moreover, human astrocytes (brain glial cells), are characterized by significantly elevated levels of both the Δ133p53α protein and the mRNA transcript [44]. An increased Δ133p53α synthesis has been shown to stimulate the proliferation of astrocytes, while the reduced level of this isoform promoted the aging process of these cells [44]. Interestingly, endogenous Δ133p53 is upregulated in human iPSC and ESC lines and Δ133p53 enhances reprograming from human fibroblasts to iPSC [45].

We applied antisense oligonucleotides targeting the 5′-terminal region of Δ133p53 mRNA aiming to inhibit translation initiation from this mRNA. It is known that accessibility to oligonucleotide hybridization does not reflect the RNA secondary structure in a simple, straightforward manner. Therefore, a comprehensive analysis of the sites accessible to oligomer hybridization was performed using the RNase H approach, based on which several antisense compounds were designed and subsequently tested in RRL translation system (Figs 4 and 5). Three selected antisense oligomers were able to reduce the level of Δ133p53 protein expressed from a plasmid in MCF-7, HepG2 and H1299 cells (Fig 6). The most effective oligomer No. 18, targeting the AUG3 codon region, reduced the amount of Δ133p53 protein by approx. 50% compared to the control cells with no oligomer added. Similar inhibition levels of 40–50% were obtained when the oligomers were used in combination as two-oligomer mixes. The best result, inhibition by 70%, was obtained when all three tested oligomers were transfected to the cells. These oligomers simultaneously targeted three regions of the 5′ terminus of Δ133p53 mRNA: two regions located just before and after the 18-nucleotide tract of adenosine residues, and the third region, in which AUG3 is embedded. These regions have not been attacked in earlier studies reported in the literature. The most effectively acting oligomer 18, which attacks the region with AUG3 translation initiation codon, seems to be of particular interest.

Several antisense oligomers and siRNAs have earlier been reported to attack the 5′-terminal region of Δ133p53 mRNA. However, almost all oligonucleotide tools have targeted only two sequence stretches of that region. The first targeted sequence spans nucleotides at positions G149 and U173 which form an unstable hairpin G152-C187 in the secondary structure model of that region (Fig 4A). In our study, antisense oligomers No. 9, 10 and 11 targeted this region of Δ133p53 mRNA. The second attacked sequence stretch involved nucleotides located between U208 and A232. In the secondary structure model this region corresponds to one strand of a double-stranded segment, which is interrupted with one- and two-nucleotide bulges and which includes an adjacent single-stranded stretch of a large internal loop. In our study antisense oligomers No. 12 and 13 hybridized to this mRNA region (Fig 4A).

Within the first G149-U173 region, siRNA has been targeted, which decreased endogenous Δ133p53α in U2OS cells, coupled to doxorubicin treatment [4]. This region has also been targeted by siRNAs for Δ133p53 silencing in two cell lines, KKU-M214R and KKU-M139R, in which apoptotic signaling has been enhanced by the upregulation of *Bax* and downregulation of *Bcl-2* [46]. A relative change (a 0.6-fold decrease) of Δ133p53, has been observed in 22Rv1 cells when siRNA targeted G149-U173 region has been used [47]. Within the second region, between U208 and A232, siRNAs have been applied in WI-38 and MRC-5 normal human fibroblasts cells. The cells have undergone a rapid and uniform senescent growth arrest showing that endogenous expression of Δ133p53 is critical for the replicative potential of normal human fibroblasts [48]. In human QSG-7701 cells, in which Δ133p53 is induced by γ-irradiation, the knockdown of Δ133p53 with siRNAs has significantly decreased the efficiencies of the three DNA DSB repair pathways, including homologous recombination, non-homologous end joining and single-strand annealing [39]. HFK and HPEC cells have been transfected with siRNAs which caused an immediate growth arrest [38]. Silencing of Δ133p53 in MCF-7 cells using siRNA targeting C214-A232 and also G155-U173 regions have significantly reduced the rate of cell proliferation [40]. Finally, morpholino antisense oligomer targeted to U308-A332 has been designed in order to specifically inhibit the expression of Δ160p53 isoform without affecting Δ133p53 or full-length p53 levels. The oligomer has been effective and specific in R273Hp53-expressing cell lines A431 and HT29 but it has failed to knock-down Δ160p53 in cell lines with different p53 mutations. The authors suggest that sequences in other regions affect the efficiency of the oligomer [5].

We also determined cell line viability upon transfection of the tested antisense oligomers (Fig 8). We chose cancerous MCF-7 cell line to observe changes in cell proliferation over time. Oligomer No. 18 as well as the mixture of the three oligomers together turned out to be effective. The antisense oligonucleotide strategy has been successfully used for decades to inhibit gene expression. Our results show that the rationally designed antisense oligomers are also attractive tools in modulation of the translation of P2-initiated p53 mRNA through attacking the 5′ terminus of the transcript. The targeted regions have not been attacked earlier and they are new places for binding antisense oligonucleotides and, possibly, also siRNAs. Upon transfection to cancerous cell lines, the studied oligomers also seem to decrease cell line viability. Thus, these oligomers can be used in further research of the role of the Δ133p53 and Δ160p53 isoforms in tumorigenesis and senescence processes.

## Supporting information

S1 File (PDF)

S1 Raw images (PDF)
