# Supplementary material for: Translation of human Δ133p53 mRNA and its targeting by antisense oligonucleotides complementary to the 5′-terminal region of this mRNA
Source: PLoS One. 2021 Sep 7;16(9):e0256938. doi: 10.1371/journal.pone.0256938 (PMC8423303; doi:10.1371/journal.pone.0256938)
Supplement: S1 File — (PDF) [file pone.0256938.s001.pdf]

Supplementary material

Translation of human Δ133p53 mRNA and its targeting by antisense oligonucleotides complementary to the 5′-terminal region of this mRNA

Paulina Żydowicz-Machtel, [1] Mariola Dutkiewicz, [1] Agata Swiatkowska, [1] Dorota Gurda-Woźna, [1] and Jerzy Ciesiołka [1]*

[1] *Institute of Bioorganic Chemistry, Polish Academy of Sciences, Noskowskiego 12/14, 61-704 Poznan, Poland*

***** Correspondence: jerzy.ciesiolka@ibch.poznan.pl (J.C.)

Supplemental Method

RNA isolation, and reverse transcription quantitative PCR (qPCR)

For RT-PCR analysis, Total RNA was isolated from Beas-2B cells using TriReagent according to the standard protocol. Reverse transcriptase reaction was performed using SuperScript IV reverse transcriptase (Thermo Fisher Scientific). The cDNA was prepared according to manufacturer's protocol from 2 µg of RNA using 50 ng of oligo(dT)$_{20}$ primer. Primers for qPCR reaction were used at a final concentration of 200 nM. Sequences of the primers were as follows:
p53 Forward: 5′-CAGATCCTAGCGTCGAGCCCC-3′,
p53 Reverse: 5′-CTGGGTCTTCAGTGAACCATTGTTC-3′,
Δ133p53 Forward: 5′-TGACTTTCAACTCTGTCTCCTTCCT-3′,
Δ133p53 Reverse: 5′-GTGCTGTGACTGCTTGTAGATGG-3′,
ß-actin Forward: 5′-AGAGCAAGAGAGGCATCCTG-3′,
ß-actin Reverse: 5′-CGACGTAGCACAGCTTCTCC-3′,
HPRT Forward: 5′-TGACCTTGATTTATTTTGCATACC-3′,
HPRT Reverse: 5′-CGAGCAAGACGTTCAGTCCT-3′.

Quantitative PCRs were performed on aliquots of cDNA samples (dilution 1:50) using 5x HOT FIREPol® EvaGreen® qPCR Mix Plus (Solis Biodyne). Datasets were collected on an Applied Biosystems™ QuantStudio™ 6 Flex Real-Time PCR System (Thermo Fisher Scientific). The cycling conditions were: 12 min at 95 °C, followed by 40 cycles consisting of

15 s at 95 °C, 20 s at 60 °C, and 20 s at 72 °C. Fluorescence signal data were collected during the 72 °C phase of each cycle. Specificity of amplified targets was assessed by melting curve analysis from 55 °C to 95 °C (in 0.5 °C increments, measuring fluorescence at each temperature) following the last cycle. The analysis showed the presence of only one specific product in each reaction.

All primer pairs were tested with regard to amplification efficiency with 4x log10 serial dilution of a random cDNA sample in triplicates. All tested primers met the criteria of efficiency 90–110% and r2 > 0.985. Statistical analysis was performed using GraphPad Prism 8. The results were expressed as a relative quantity according to the equation RQ=2ΔΔCt ± standard deviation (SD).

Supplemental Figures and Tables

Supplemental Figure S1. Secondary structure mapping of the 5′-terminal region of Δ133p53 mRNA by Pb^{2+}-induced cleavage and SHAPE methods and secondary structure prediction. A) Autoradiograms show electrophoretic separation of products after the reactions with Pb^{2+} ions analyzed on 12% polyacrylamide gels. A, T, G, C indicate sequencing lines, 0.5, 1 and 2 are the concentrations of Pb^{2+} ions, 0 is a control reaction in which no Pb^{2+} was added. Selected nucleotide residues are labeled on the right. B) Autoradiograms of electrophoretic separation of products after SHAPE analyzed on 12% polyacrylamide gels. A, T, G, C indicate sequencing lines, (NMIA +) denotes reaction with a modifying reagent, and (-) means a reaction with the control to which NMIA was not added. On the right, the selected positions of nucleotide residues are indicated. C) RNA secondary structure model prediction probability of 5′-terminal region of Δ133p53 mRNA. The probability reflects the odds of being in the unspecified pair, for a paired nucleotide, or of being single-stranded, for an unpaired nucleotide. The predicted secondary structure was also color annotated to indicate these probabilities. The higher the probability, the higher the confidence of the prediction accuracy. The color of the letters corresponds to the probability values calculated by RNAstructure5.7, according to the key in the figure. Motifs marked in red, orange and yellow have the highest probability of occurrence in the analyzed RNA fragment, so they are considered structurally well determined.

Supplemental Figure S2. Western blot analysis of Δ133p53-FLAG expression in H1299 cell line transfected or non-transfected with Δ133p53-FLAG expression vector. Left panel shows analysis with the use of FLAG antibody, right panel with Pab421 antibody. Left image on each panel presents a membrane with protein ladder, right image shows the same membrane after 2 minute visualization.

Supplemental Figure S3. MCF7 cells viability 24, 48 and 72 h after transfection with antisense oligonucleotides targeting the 5′-terminal region of Δ133p53 mRNA estimated *via* flow cytometry. Viability of MCF7 cells was evaluated by dual staining with Calcein/Ethidium Bromide. The percentage of live (lower right quadrant) and dead (upper left and upper right quadrant) cells is presented in the diagram as the mean of the total analyzed population (10 000 cells)) ± SD from at least three independent experiments. The presented data are non-normalized and representative for each experimental condition.

Supplemental Figure S4. Relative expression of Δ133p53-FLAG mRNAs in H1299, HepG2 and MFC-7 cell lines after transfection of the Δ133p53-FLAG expression vector and antisense oligonucleotides determined by real-time PCR.

Supplemental Table ST1. Numerical SHAPE raw data.

Supplemental Table ST2. Antisense oligonucleotides used in this study. The oligonucleotides highlighted in yellow were synthesized as their 2′*O*-methylated derivatives with phosphorothioate internucleotide bonds.

Fig. S1

A

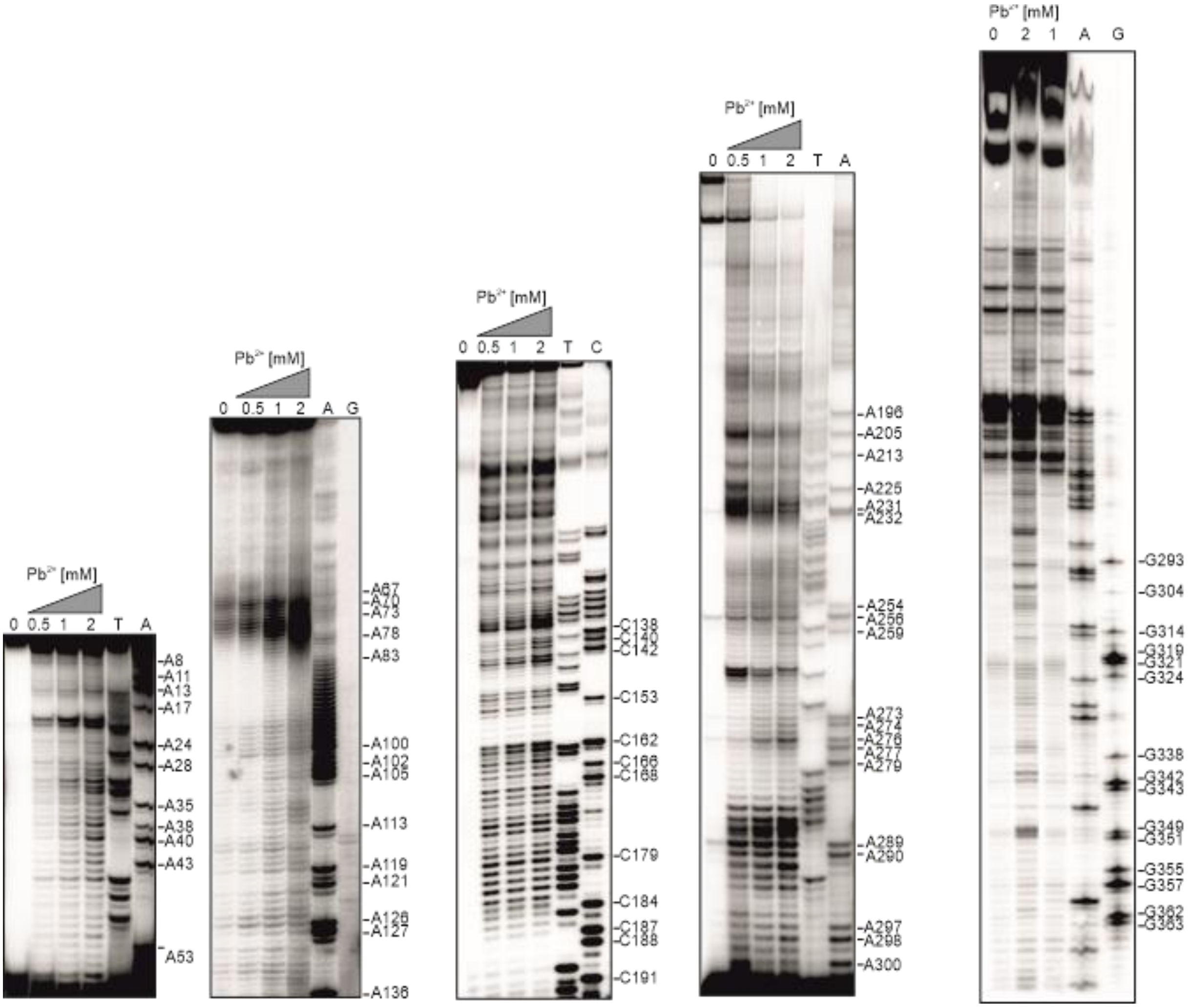

Pb2+ [mM]
0 0.5 1 2 T A
A8
A11
A13
A17
A24
A28
A35
A38
A40
A43
A53
Pb2+ [mM]
0 0.5 1 2 A G
A67
A70
A73
A78
A83
A100
A102
A105
A113
A119
A121
A126
A127
A136
Pb2+ [mM]
0 0.5 1 2 T C
C138
C140
C142
C153
C162
C166
C168
C179
C184
C187
C188
C191
Pb2+ [mM]
0 0.5 1 2 T A
A196
A205
A213
A225
A231
A232
A254
A256
A259
A273
A274
A276
A277
A279
A289
A290
A297
A298
A300
Pb2+ [mM]
0 2 1 A G
G293
G304
G314
G319
G321
G324
G338
G342
G343
G349
G351
G355
G357
G362
G363

B

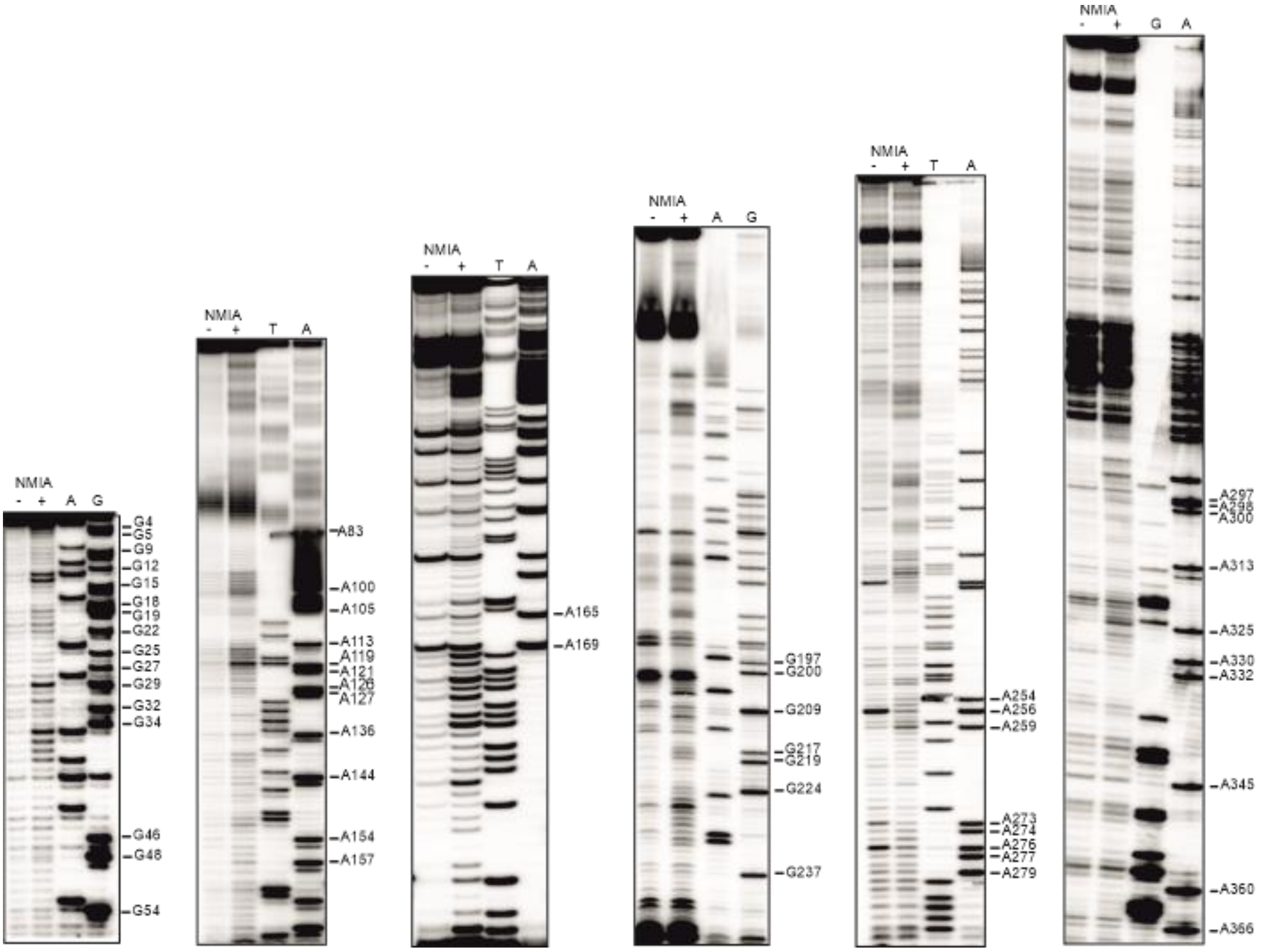

NMIA
- + A G
G4
G5
G9
G12
G15
G18
G19
G22
G25
G27
G29
G32
G34
G46
G48
G54
NMIA
- + T A
A83
A100
A105
A113
A119
A121
A126
A127
A136
A144
A154
A157
NMIA
- + T A
A165
A169
NMIA
- + A G
G197
G200
G209
G217
G219
G224
G237
NMIA
- + T A
A254
A256
A259
A273
A274
A276
A277
A279
NMIA
- + G A
A297
A298
A300
A313
A325
A330
A332
A345
A360
A366

Fig. S1 C

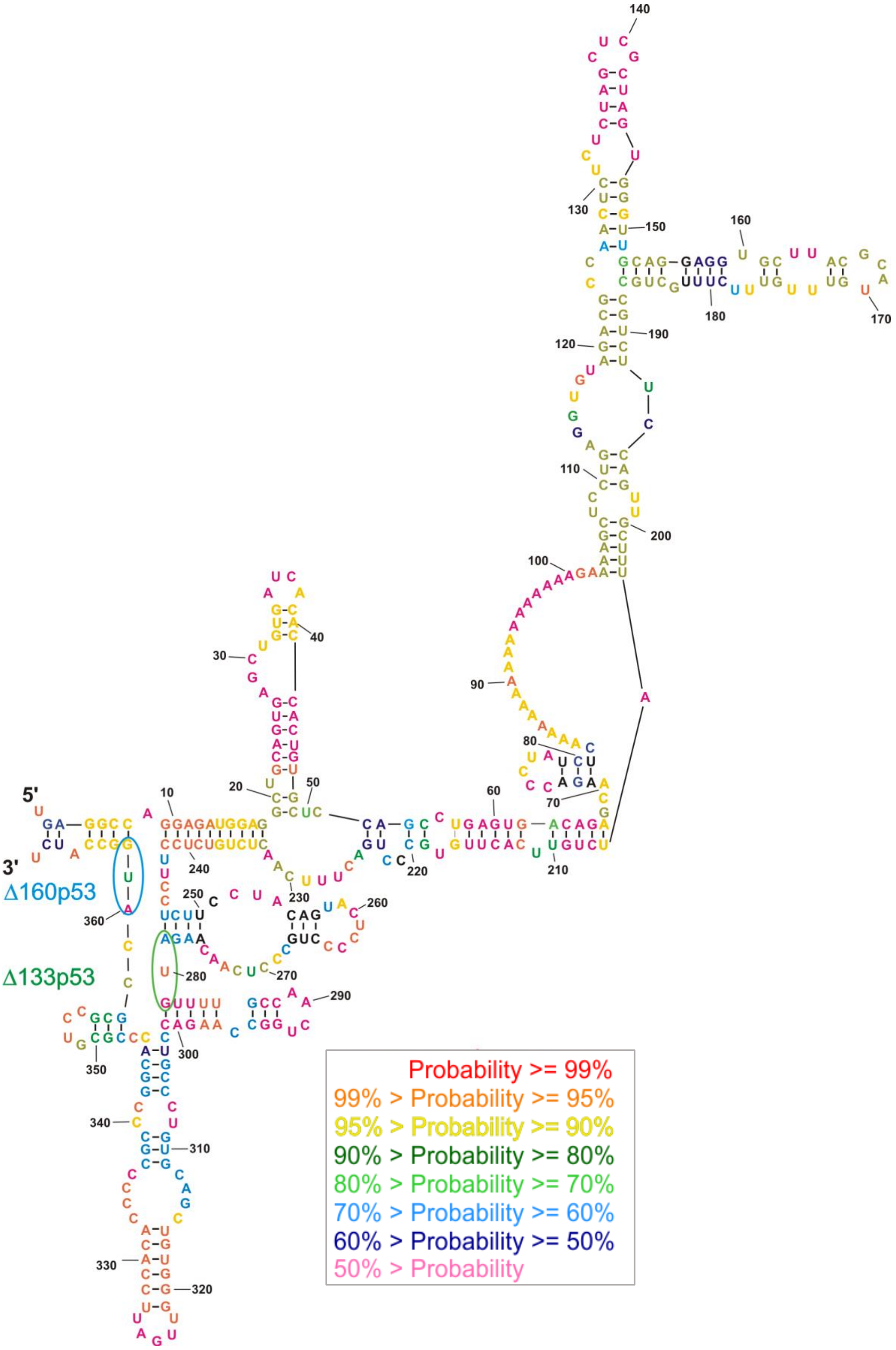
5'
3'
Δ160p53
Δ133p53
Probability >= 99%
99% > Probability >= 95%
95% > Probability >= 90%
90% > Probability >= 80%
80% > Probability >= 70%
70% > Probability >= 60%
60% > Probability >= 50%
50% > Probability

Fig. S2

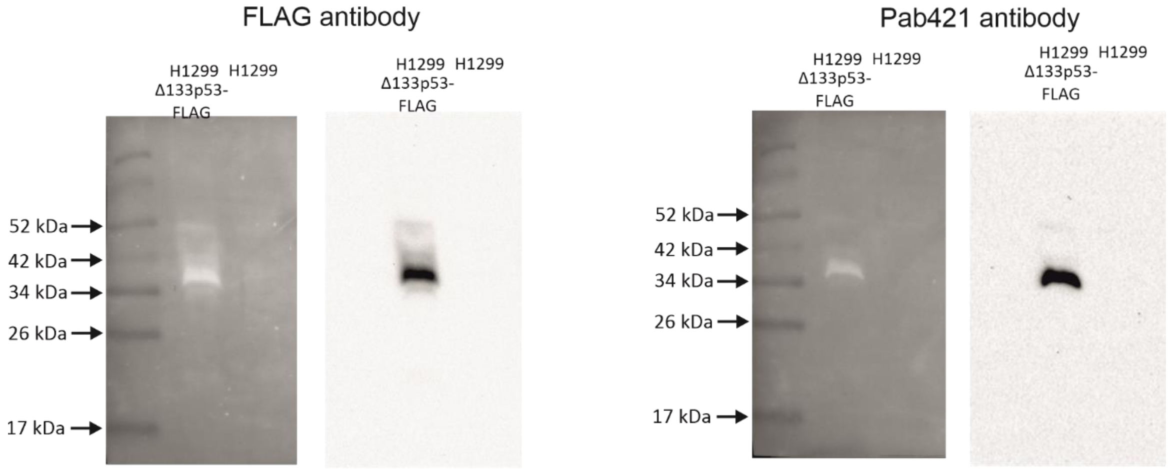
FLAG antibody
H1299 Δ133p53-FLAG
H1299
H1299 Δ133p53-FLAG
H1299
52 kDa
42 kDa
34 kDa
26 kDa
17 kDa
Pab421 antibody
H1299 Δ133p53-FLAG
H1299
H1299 Δ133p53-FLAG
H1299
52 kDa
42 kDa
34 kDa
26 kDa
17 kDa

Fig. S3

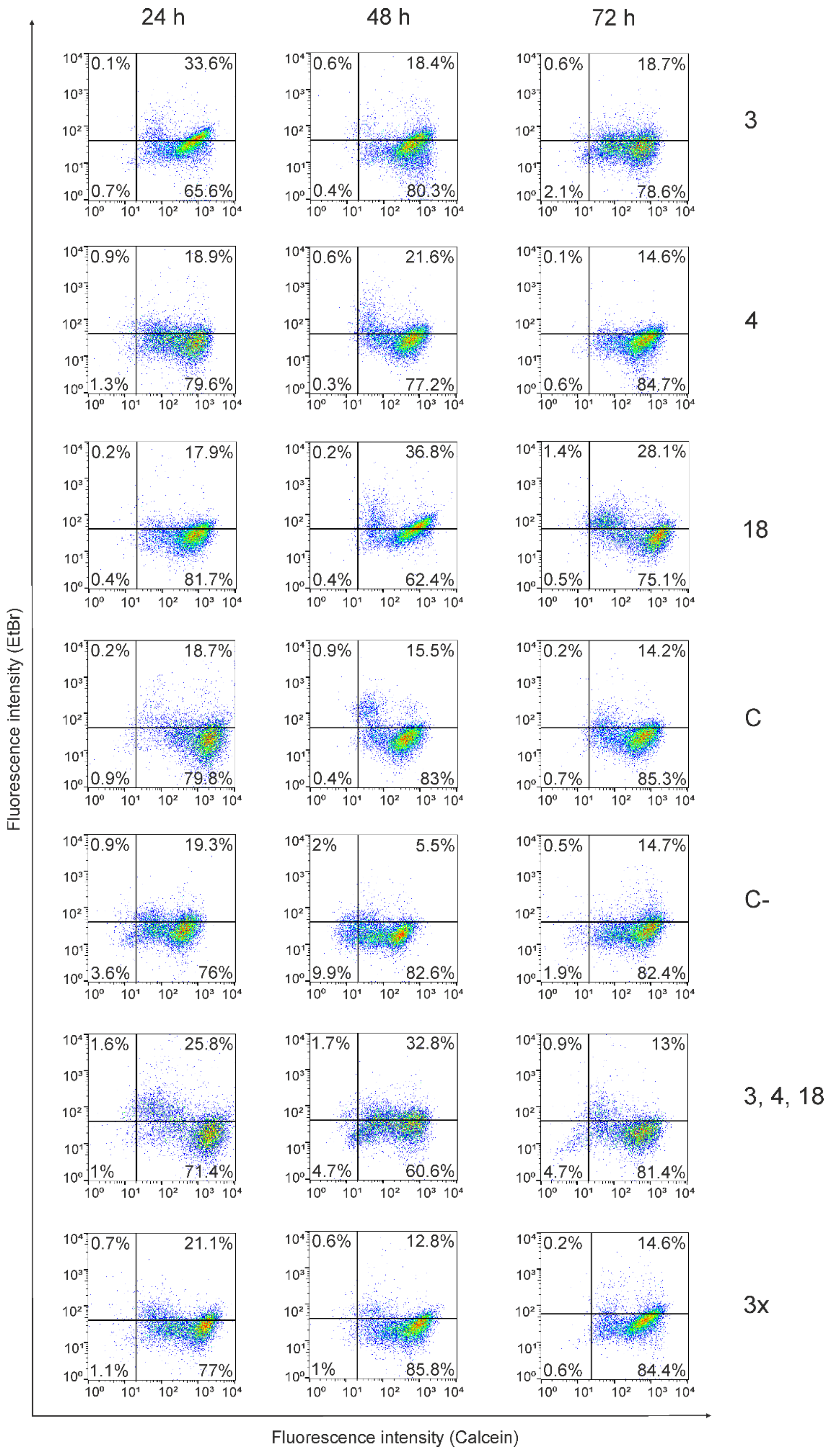
24 h
48 h
72 h
0.1% 33.6% 0.7% 65.6%
0.6% 18.4% 0.4% 80.3%
0.6% 18.7% 2.1% 78.6%
3
0.9% 18.9% 1.3% 79.6%
0.6% 21.6% 0.3% 77.2%
0.1% 14.6% 0.6% 84.7%
4
0.2% 17.9% 0.4% 81.7%
0.2% 36.8% 0.4% 62.4%
1.4% 28.1% 0.5% 75.1%
18
0.2% 18.7% 0.9% 79.8%
0.9% 15.5% 0.4% 83%
0.2% 14.2% 0.7% 85.3%
C
0.9% 19.3% 3.6% 76%
2% 5.5% 9.9% 82.6%
0.5% 14.7% 1.9% 82.4%
C-
1.6% 25.8% 1% 71.4%
1.7% 32.8% 4.7% 60.6%
0.9% 13% 4.7% 81.4%
3, 4, 18
0.7% 21.1% 1.1% 77%
0.6% 12.8% 1% 85.8%
0.2% 14.6% 0.6% 84.4%
3x
Fluorescence intensity (EtBr)
Fluorescence intensity (Calcein)

Fig. S4.

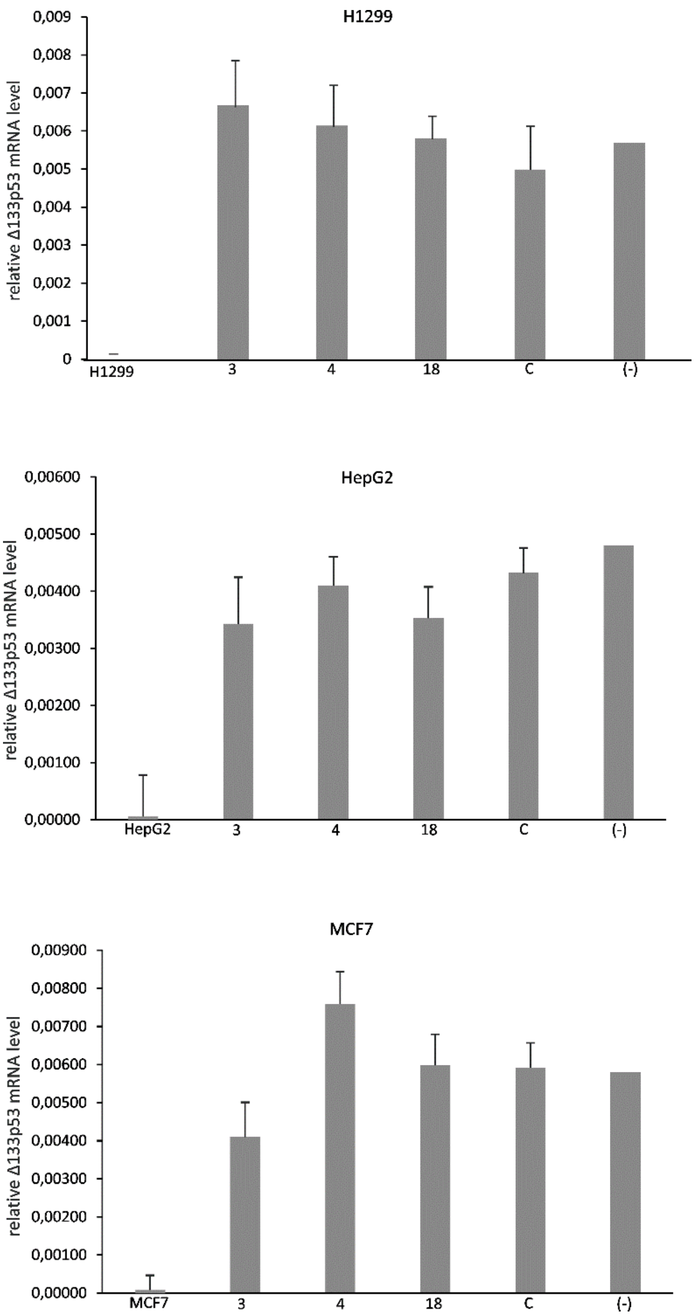
H1299
relative Δ133p53 mRNA level
0,009
0,008
0,007
0,006
0,005
0,004
0,003
0,002
0,001
0
H1299
3
4
18
C
(-)
HepG2
relative Δ133p53 mRNA level
0,00600
0,00500
0,00400
0,00300
0,00200
0,00100
0,00000
HepG2
3
4
18
C
(-)
MCF7
relative Δ133p53 mRNA level
0,00900
0,00800
0,00700
0,00600
0,00500
0,00400
0,00300
0,00200
0,00100
0,00000
MCF7
3
4
18
C
(-)

Supplemental Table ST1. Numerical SHAPE raw data.

seqnum	seq	reactivity	RX,area	BG,area	normalization
1	U	0			
2	G	0			
3	A	0			
4	G	7882,18	20600,66	12718,48	0.4717461871678
5	G	9415,13	16484,73	7069,6	0.5634928001123
6	C	6542,61	12896,51	6353,9	0.3915733111431
7	C	2828,9	8348,3	5519,4	0.169308844619
8	A	3775,18	10367,62	6592,44	0.2259434281978
9	G	3774,54	9808,96	6034,42	0.2259051243834
10	G	3781,36	8834,84	5053,48	0.2263132994056
11	A	4908,27	9259,72	4351,45	0.2937585361017
12	G	5754,98	9819,64	4064,66	0.3444338840558
13	A	19410,36	26977,54	7567,18	1.1617044169956
14	U	24329,51	30519,44	6189,93	1.4561141179421
15	G	7290,48	10626,63	3336,15	0.4363331137608
16	G	4915,38	9292,98	4377,6	0.2941840675398
17	A	2582,4	5590,01	3007,61	0.154555891104
18	G	4475,86	8674,21	4198,35	0.2678789230006
19	G	9322,54	15107,95	5785,41	0.5579513154634
20	C	7760,96	12757,72	4996,76	0.4644912053216
21	U	8996,53	19271,74	10275,21	0.5384397115063
22	G	7029,45	14070,36	7040,91	0.4207105439595
23	C	3736,83	9054,16	5317,33	0.2236481918193
24	A	2002,2	6484,13	4481,93	0.119831089362
25	G	3083,79	7651,69	4567,9	0.1845639372009
26	U	3947,58	9414,35	5466,77	0.2362615182018
27	G	2721,52	6069,38	3347,86	0.1628821827592
28	A	8828,38	14478,78	5650,4	0.5283759827698
29	G	32623,29	41926,44	9303,15	1.9524944457459
30	C	8392,85	14785,14	6392,29	0.5023096385735
31	U	15079,58	20155,7	5076,12	0.9025084899218
32	G	3707,36	10633,25	6925,89	0.2218844208656
33	U	3483,62	10758,67	7275,05	0.2084936467502
34	G	6864,87	10733,85	3868,98	0.4108604786877
35	A	33556,59	40774,96	7218,37	2.0083521800889
36	U	23438,83	27803,36	4364,53	1.4028071782393
37	C	16683,34	21368,72	4685,38	0.9984930608314
38	A	17659,42	22912,34	5252,92	1.0569111657682
39	C	5661,95	9491,48	3829,53	0.3388660655345
40	A	3160,21	14392,2	11231,99	0.1891376520391
41	C	1505,13	4754,81	3249,68	0.0900815940123
42	C	3039,59	6555,56	3515,97	0.1819185800189

43	A	3446,89	7776,97	4330,08	0.2062953669019
44	C	2893,9	12607,55	9713,65	0.173199075769
45	U	6465,9	14155,04	7689,14	0.386982239889
46	G	2925,45	7188,21	4262,76	0.1750873341195
47	U	5180,44	11969,26	6788,82	0.3100478316724
48	G	2456,14	6237	3780,86	0.1469992667194
49	C	2734,96	5620,71	2885,75	0.1636865628616
50	U	3108,34	7065,87	3957,53	0.1860332475814
51	C	1908,35	6371,35	4463	0.1142141940785
52	C	1726,02	6920,26	5194,24	0.1033017964542
53	A	3362,59	7915,42	4552,83	0.2012500363489
54	G	3687,72	11751,17	8063,45	0.2207089725612
55	C	4294	9783,96	5489,96	0.25699465474
56	C	2558,84	11990,57	9431,73	0.1531458319364
57	U	2281,65	8929,65	6648	0.1365560908215
58	G	0			
59	A	0			
60	G	0			
61	U	0			
62	G	0			
63	A	0			
64	C	0			
65	A	0			
66	G	0			
67	A	0			
68	G	0			
69	C	0			
70	A	0			
71	A	0			
72	G	0			
73	A	0			
74	C	0			
75	C	0			
76	C	0			
77	U	0			
78	A	0			
79	U	0			
80	C	0			
81	U	0			
82	C	0			
83	A	0			
84	A	0			
85	A	0			
86	A	0			

87	A	0			
88	A	0			
89	A	0			
90	A	0			
91	A	0			
92	A	0			
93	A	0			
94	A	0			
95	A	0			
96	A	0			
97	A	0			
98	A	0			
99	A	0			
100	A	0			
101	G	0			
102	A	0			
103	A	0			
104	A	0			
105	A	0			
106	G	513,08	733,85	220,77	0.0307076892068
107	C	267,43	431,03	163,6	0.0160056079453
108	U	205,51	360,19	154,68	0.0122997139021
109	C	172,35	328	155,65	0.0103150975185
110	C	3152,51	4339,94	1187,43	0.1886768092721
111	U	1371,29	3447,29	2076	0.0820713088259
112	G	2770,28	5494,39	2724,11	0.1658004546188
113	A	8302,16	11879,06	3576,9	0.4968818683736
114	G	19416,72	24614,31	5197,59	1.1620850611512
115	G	18934,01	24604,44	5670,43	1.1331950076371
116	U	20752,3	24008,31	3256,01	1.242019136833
117	G	7832,17	9996,43	2164,26	0.4687531031707
118	U	17944,14	23230,3	5286,16	1.0739515751994
119	A	19688,6	22953,29	3264,69	1.178357000306
120	G	10530,85	13470,59	2939,74	0.6302683185535
121	A	7070,63	10107,29	3036,66	0.4231751550173
122	C	7995,76	11023,43	3027,67	0.4785439172296
123	G	3379,9	6795,08	3415,18	0.202286034829
124	C	8404,49	11498,44	3093,95	0.5030062891979
125	C	5158,28	7165,87	2007,59	0.3087215620988
126	A	6488,27	11540,58	5052,31	0.3883210779017
127	A	5204,22	8346,59	3142,37	0.3114710577762
128	C	4504,97	6136,07	1631,1	0.2696211480587
129	U	1646,63	3416,26	1769,63	0.0985503279773
130	C	2495,87	4316,52	1820,65	0.1493770956977

131	U	2936,62	4076,32	1139,7	0.1757558553802
132	C	7668,18	10128,27	2460,09	0.4589383492278
133	U	3268,84	5495,01	2226,17	0.1956391260364
134	C	1844,15	3486,43	1642,28	0.1103718426965
135	U	3179,34	4749,72	1570,38	0.1902825769914
136	A	5616,69	10404,39	4787,7	0.3361572676599
137	G	3721,67	6264,24	2542,57	0.2227408702157
138	C	1595,69	3096,19	1500,5	0.0955015837499
139	U	2974,72	4746,04	1771,32	0.1780361293312
140	C	11233,59	14241,37	3007,78	0.6723271037589
141	G	10058,65	13824,51	3765,86	0.6020072854915
142	C	4965,31	6984,92	2019,61	0.2971723635601
143	U	3189,79	5781,18	2591,39	0.1909080064609
144	A	5877,2	8647,51	2770,31	0.351748715612
145	G	8994,58	11516,93	2522,35	0.5383230045718
146	U	4596,47	6059,97	1463,5	0.2750973965237
147	G	5612,95	8138,74	2525,79	0.3359334297445
148	G	4278,4	6368,45	2090,05	0.256060999264
149	G	2333,36	3536,17	1202,81	0.1396509193256
150	U	4445,01	6840,63	2395,62	0.2660325594471
151	U	11208,19	14142,68	2934,49	0.6708069211249
152	G	13603,67	16733,75	3130,08	0.8141757044357
153	C	7463,87	9885,58	2421,71	0.4467104549777
154	A	2736,96	6255,7	3518,74	0.1638062622816
155	G	5154,91	8086,77	2931,86	0.3085198685761
156	G	5667,96	7306,79	1638,83	0.3392257622916
157	A	11455,5	14132	2676,5	0.685608352905
158	G	6337,18	9687,66	3350,48	0.3792783852178
159	G	8187,41	11677,13	3489,72	0.4900141141511
160	U	0			
161	G	0			
162	C	0			
163	U	0			
164	U	0			
165	A	4407,63	13987,2	9579,57	0.2637953772873
166	C	4871,48	7503,22	2631,74	0.2915566652708
167	G	2543,36	6731,83	4188,47	0.1522193584256
168	C	249,83	4229,01	3979,18	0.0149522530493
169	A	22058,49	51754,54	29696,05	1.3201942295379
170	U	29621,74	38607,1	8985,36	1.7728525486954
171	G	14539,18	20742,59	6203,41	0.8701657066378
172	U	6779,28	9514,68	2735,4	0.4057379420088
173	U	43579,41	53535,65	9956,24	2.6082150504711
174	U	18408,16	23117,01	4708,85	1.1017230376336

175	G	30214,36	39348,69	9134,33	1.8083206838356
176	U	5584,47	7893,92	2309,45	0.3342289100037
177	U	28423,27	33142,04	4718,77	1.7011244667517
178	U	49800,35	55886,76	6086,41	2.9805365053985
179	C	4393,08	12891,18	8498,1	0.2629245640068
180	U	5694,49	11318,47	5623,98	0.3408135750979
181	U	5663,56	8613,93	2950,37	0.3389624235676
182	U	8537,86	12366,16	3828,3	0.5109884450206
183	G	21259,28	29566,47	8307,19	1.2723617428088
184	C	6671,75	8999,62	2327,87	0.3993023026925
185	U	1891,27	6762,23	4870,96	0.1131919610317
186	G	5250,64	7510,26	2259,62	0.3142492813144
187	C	6959,1	8925,87	1966,77	0.416500116861
188	C	1565,83	2568,76	1002,93	0.0937144714093
189	G	11427,72	13757,42	2329,7	0.6839457279612
190	U	576,52	5564,37	4987,85	0.0345045548092
191	C	569,26	5955,87	5386,61	0.0340700459146
192	U	704,72	4668,23	3963,51	0.0421772876312
193	U	-166,44	3625,89	3792,33	
194	C	1323,15	6147,12	4823,97	0.0791901437865
195	C	2706,48	5903,5	3197,02	0.1619820431208
196	A	-999,01	4397,58	5396,59	
197	G	-1458,81	3924,54	5383,35	
198	U	-346,53	5491,2	5837,73	
199	U	2936,24	6034,26	3098,02	0.1757331124904
200	G	-130,64	8802,45	8933,09	
201	C	-4348,78	10631,58	14980,36	
202	U	2684,3	9770,58	7086,28	0.160654576553
203	U	2289,62	7775,84	5486,22	0.1370330930102
204	U	3447,18	14015,69	10568,51	0.2063127233178
205	A	8112,65	13697,76	5585,11	0.4855397498315
206	U	6581,51	15472,84	8891,33	0.3939014648621
207	C	12770,06	18523,97	5753,91	0.7642843876826
208	U	5133,14	10352,9	5219,76	0.3072169403894
209	G	-209,52	8561,4	8770,92	
210	U	2197,04	7226,86	5029,82	0.1314922068584
211	U	2260,44	10112,64	7852,2	0.1352866784724
212	C	6461,23	10874,64	4413,41	0.3867027417433
213	A	5930,99	9602,28	3671,29	0.3549680315129
214	C	-302,06	6542,79	6844,85	
215	U	1675,82	6921,01	5245,19	0.1002973410122
216	U	1195,37	7366	6170,63	0.0715425478427
217	G	2221,82	9568,2	7346,38	0.1329752826722
218	U	3758,93	10824,38	7065,45	0.2249708704103

219	G	10408,77	13469,22	3060,45	0.6229618659567
220	C	4426,77	9510,61	5083,84	0.2649409007367
221	C	4889,22	8527,05	3637,83	0.2926183991262
222	C	2356,45	4852,49	2496,04	0.1410328491295
223	U	339,97	5870,26	5530,29	0.0203471059087
224	G	2343,6	6344,81	4001,21	0.140263780356
225	A	800,61	14191,75	13391,14	0.0479162763231
226	C	8870,81	14440,63	5569,82	0.5309154059651
227	U	7109,66	16817,72	9708,06	0.4255110891986
228	U	3384,56	10599,4	7214,84	0.2025649344776
229	U	5784,44	20288,64	14504,2	0.3461970565124
230	C	16653,08	30483,45	13830,37	0.9966820086068
231	A	14817,66	19512,17	4694,51	0.8868326538786
232	A	1626,26	14711,69	13085,43	0.0973311893846
233	C	10055,18	14686,83	4631,65	0.6017996069978
234	U	10184,79	18544,69	8359,9	0.6095567279109
235	C	4196,64	9174,99	4978,35	0.2511676869744
236	U	-765,42	10288,33	11053,75	
237	G	221,26	5378,9	5157,64	0.0132423468346
238	U	-3271,76	10665,1	13936,86	
239	C	396,07	4786,6	4390,53	0.0237046746397
240	U	976,74	3435,35	2458,61	0.0584576057454
241	C	-846,98	5256,41	6103,39	
242	C	-61,13	7409,89	7471,02	
243	U	1125,67	3877,03	2751,36	0.0673710230557
244	U	1398,37	4536,2	3137,83	0.0836920389727
245	C	381,97	16057,95	15675,98	0.0228607937287001
246	C	6156,19	10201,05	4044,86	0.3684461862049
247	U	1478,46	8795,45	7316,99	0.0884854022466001
248	C	2579,19	15720,93	13141,74	0.1543637735349
249	U	2295,37	5688,96	3393,59	0.1373772288427
250	U	2176,69	5879,18	3702,49	0.1302742652599
251	C	6339,77	12971,58	6631,81	0.3794333959667
252	C	7441,65	20415,45	12973,8	0.4453805944215
253	U	2006,43	6100,57	4094,14	0.1200842536353
254	A	2601,84	12416,95	9815,11	0.1557193694664
255	C	22169,31	42372,39	20203,08	1.3268267744001
256	A	58620,3	92045,7	33425,4	3.508407955113
257	G	944,49	3368,13	2423,64	0.0565274525979
258	U	1217,01	4279,25	3062,24	0.0728376955671
259	A	2323,75	5368,1	3044,35	0.1390757636125
260	C	1867,25	3903,78	2036,53	0.1117543709975
261	U	970,8	3185,6	2214,8	0.058102098468
262	C	836,06	2165,74	1329,68	0.0500379485426

263	C	1212,98	2702,03	1489,05	0.0725965012358
264	C	932,93	2740	1807,07	0.0558355899503
265	C	332,09	2678,4	2346,31	0.0198754901939
266	U	264,45	2598,59	2334,14	0.0158272558095
267	G	1023,85	2536,05	1512,2	0.0612771255835
268	C	2126,12	3891,45	1765,33	0.1272476654252
269	C	1001,86	2341,93	1340,07	0.0599610304606
270	C	1073,21	2411,53	1338,32	0.0642313072691
271	U	827,77	2764,73	1936,96	0.0495417944467
272	C	927,73	2557,99	1630,26	0.0555243714583
273	A	946,01	3742,25	2796,24	0.0566184241571
274	A	433,55	4536,01	4102,46	0.0259478417705
275	C	2188,61	7327,05	5138,44	0.1309876738031
276	A	762,85	8023,8	7260,95	0.0456563512735
277	A	1390,41	6429,56	5039,15	0.0832156352811
278	G	2328,64	6428,11	4099,47	0.1393684286944
279	A	2302,37	6311,97	4009,6	0.1377961768127
280	U	1960,65	5808,16	3847,51	0.1173443339115
281	G	1982,18	6485,02	4502,84	0.1186328981678
282	U	1098,23	5136,55	4038,32	0.0657287470133
283	U	1734,85	6088,65	4353,8	0.1038302693935
284	U	717,61	2441,31	1723,7	0.0429487503931
285	U	529,14	4866,36	4337,22	0.0316688755494
286	G	2360,31	7504,05	5143,74	0.1412638690101
287	C	1722,38	5832,6	4110,22	0.1030839435098
288	C	3370,72	11272,55	7901,83	0.2017366144912
289	A	2921,17	8948,34	6027,17	0.1748311773607
290	A	3577,74	6853,96	3276,22	0.2141267014554
291	C	3890,25	7266,79	3376,54	0.2328303343275
292	U	3084,31	4825,48	1741,17	0.1845950590501
293	G	3163,32	6522,47	3359,15	0.1893237846372
294	G	2031,83	4715,28	2683,45	0.1216044362693
295	C	1287,35	4240,1	2952,75	0.0770475241685
296	C	680,3	5376,96	4696,66	0.040715757713
297	A	1848,82	2613,28	764,46	0.1106513408422
298	A	3005,19	3596,21	591,02	0.1798597499949
299	G	2496,03	2919,44	423,41	0.1493866716513
300	A	1245,25	1559,57	314,32	0.0745278513775
301	C	505,51	715,87	210,36	0.0302546269021
302	C	329,27	524,22	194,95	0.0197067140117
303	U	346,93	639,89	292,96	0.0207636598903
304	G	522,45	875,94	353,49	0.0312684809895
305	C	541,85	795,29	253,44	0.0324295653635
306	C	450,09	690,38	240,29	0.0269377559739

307	C	411,11	759,53	348,42	0.0246048142781
308	U	342,98	724,11	381,13	0.0205272535358
309	G	553,77	954,66	400,89	0.0331429739067
310	U	687,88	1058,67	370,79	0.0411694185148
311	G	488,48	861,87	373,39	0.0292353863408
312	C	144,28	729,8	585,52	0.0086351161588
313	A	227,43	766,08	538,65	0.0136116195453
314	G	730,65	1695,05	964,4	0.0437291906115
315	C	1260,97	2027,69	766,72	0.0754686888187
316	U	1719,99	2458,45	738,46	0.1029409027029
317	G	1826,21	2337,05	510,84	0.1092981388991
318	U	1754,29	2336,35	582,06	0.1049937477559
319	G	1373,24	2054,91	681,67	0.0821880157604
320	G	1026,57	1534,09	507,52	0.0614399167947
321	G	452,89	925,8	472,91	0.0271053351619
322	U	1536,42	2207,94	671,52	0.0919542914382
323	U	2053,04	2959,79	906,75	0.1228738486184
324	G	3719,88	4821,82	1101,94	0.2226337392348
325	A	3276,04	4363,42	1087,38	0.1960700439484
326	U	2836,16	3645,74	809,58	0.1697433535136
327	U	3146,09	4018,35	872,26	0.1882925741339
328	C	2692,63	3387,41	694,78	0.1611531246373
329	C	1116,8	2277,59	1160,79	0.066840156128
330	A	92,99	1351,15	1258,16	0.0055654245329
331	C	49,01	1255	1205,99	0.0029332342871
332	A	5,85	911,14	905,29	0.0003501208035
333	C	219,4	785,1	565,7	0.013131026374
334	C	186,4	605,76	419,36	0.011155985944
335	C	65,43	527,28	461,85	0.0039159665253
336	C	110,24	648,9	538,66	0.0065978320304
337	C	206,35	821,71	615,36	0.0123499876585
338	G	229,59	763,22	533,63	0.0137408949189
339	C	278,03	747,37	469,34	0.0166400148713
340	C	484,19	1025,46	541,27	0.0289786310849
341	C	1483	2294,61	811,61	0.08875711993
342	G	1387,36	2223,84	836,48	0.0830330936656
343	G	1273,86	2027,41	753,55	0.0762401515806
344	C	556,62	885,81	329,19	0.0333135455802
345	A	31,36	370,25	338,89	0.0018768869056
346	C	-92,86	402,85	495,71	
347	C	113,25	662,37	549,12	0.0067779796575
348	C	676,77	1366,11	689,34	0.0405044882367
349	G	969,57	1905,59	936,02	0.0580284833247
350	C	2018,17	3688,43	1670,26	0.1207868892307

351	G	3095,62	4457,52	1361,9	0.1852719592702
352	U	2654,36	3511,94	857,58	0.1588626762356
353	C	2187,41	2855,86	668,45	0.1309158541511
354	C	838,04	1186,38	348,34	0.0501564509684
355	G	966,61	1437,45	470,84	0.0578513281831
356	C	878,65	1382,93	504,28	0.0525869476915
357	G	967,86	1671,45	703,59	0.0579261403206
358	C	629,01	1613,69	984,68	0.0376460660871
359	C	771,69	2356,25	1584,56	0.0461854227099
360	A	790,35	2335,43	1545,08	0.0473022182985
361	U	2170,53	3550,55	1380,02	0.1299055910463
362	G	2471,99	3420,38	948,39	0.1479478846229

Supplemental Table ST2. Antisense oligonucleotides used in this study. The oligonucleotides highlighted in yellow were synthesized as their 2'*O*-methylated derivatives with phosphorothioate internucleotide bonds.

oligo no	sequence	length	oligo-oligo energy	oligo self energy	overall energy	duplex energy	Tm	location in mRNA	2'ome PS
1	GATCACAGCTCACTGCAGCC	20	-8,8	-1,4	-13,9	27,8	76	18	
2	GCTGGAGCACAGTGGTGTGAT	21	-9,3	-2,2	-14,4	-28,2	79,2	35	
3	TTGAGATAGGGTCTTGCTCTGT	22	-6,6	-0,2	-20,2	-27	76,2	63	UUGAGAUAGGGUCUUGCUCUGU
4	TACACCTCAGGAGCTTTTCTT	21	-6,9	-1,3	-14,5	-25,9	73,7	99	UACACCUCAGGAGCUUUUCUU
5	TTGGCGTCTACACCTCAGGA	20	-5,1	0	-12,9	-27	76	108	
6	AGAGAGTTGGCGTCTACACCT	21	-6	0	-15,8	-27,4	76	113	
7	ACTAGCGAGCTAGAGAGAGTTGG	23	-10,5	-0,8	-19,6	-27,4	73,1	124	
8	AACCCACTAGCGAGCTAGAGA	21	-10,5	-0,8	-18,9	-26,7	71,5	131	
9	GAAACAAACATGCGTAAGCACCTC	24	12,2	-5,8	-15	-26	64,2	156	
10	CAAAGAAACAAACATGCGTAAGCACC	26	-11,6	-5,8	-14,8	-25,8	60,8	158	
11	GCAGCAAAGAAACAAACATGCGTAAG	26	-7,7	-1,5	-13,8	-25,4	60,7	162	
12	GTCAGGGCACAAGTGAACAGATAA	24	-5,3	0	-14	-26,6	68,2	203	
13	TGAAAGTCAGGGCACAAGTGAACAGA	26	-5,3	0	-18,1	-28,6	68,5	206	
14	GGAAGGAGACAGAGTTGAAAGTCAGG	26	-7,2	-1,8	-14,2	-28,4	69,6	221	
15	ACTGTAGGAAGAGGAAGGAGACAGAG	26	-4,8	-0,7	-13,7	-28,1	69,2	233	
16	GGGAGTACTGTAGGAAGAGGAAG	23	-6	0	-19,5	-25,4	68,8	242	
17	ACCAGGGCGUAUCUCUCCAUAG	22	-5,4	-1,1	-16,5	-25,9	70,2	263	
18	AGTTGGCAAAACATCTTGTTGAGGG	25	-10,5	-1,3	-15,8	-28	69,6	268	AGUUGGCAAAACAUCUUGUUGAGGG
19	GGCCAGTTGGCAAAACATCTTG	22	-9,6	-1,7	-14,4	-26,7	70,1	275	
C									ACCAGGGCGUAUCUCUCCAUA
