# Supplementary material for: Translation of human Δ133p53 mRNA and its targeting by antisense oligonucleotides complementary to the 5′-terminal region of this mRNA
Source: PLoS One. 2021 Sep 7;16(9):e0256938. doi: 10.1371/journal.pone.0256938 (PMC8423303; doi:10.1371/journal.pone.0256938)

Full-length images:

Figure 3a: method - radioactivity scanning

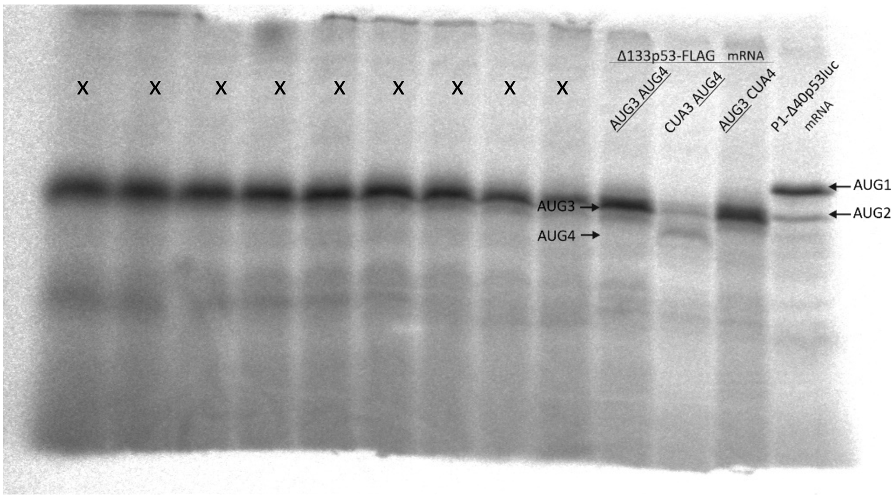

Figure 3B: method - chemiluminescence digitalization

Δ133p53-FLAG

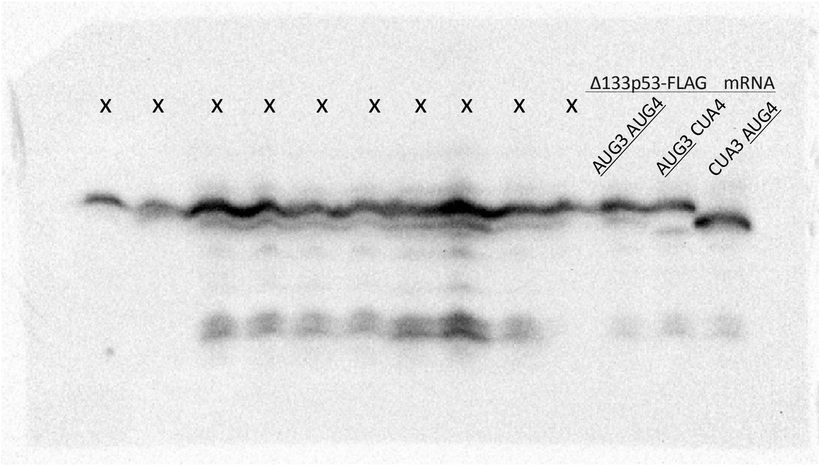

p53

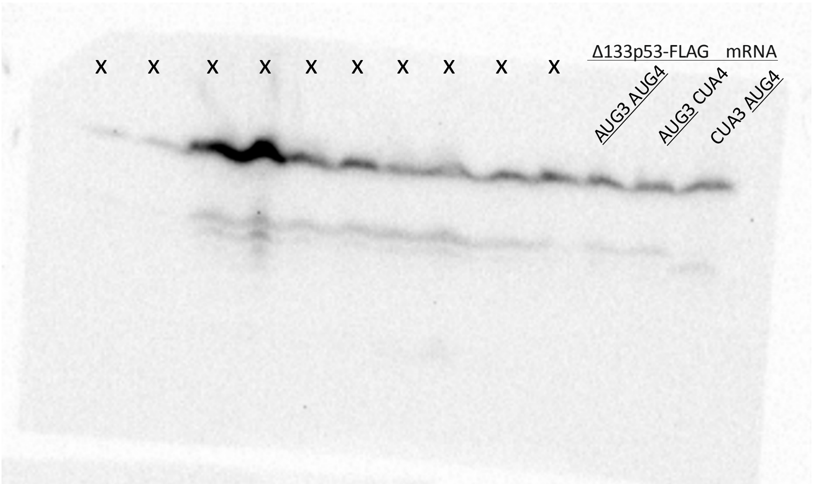

GAPDH

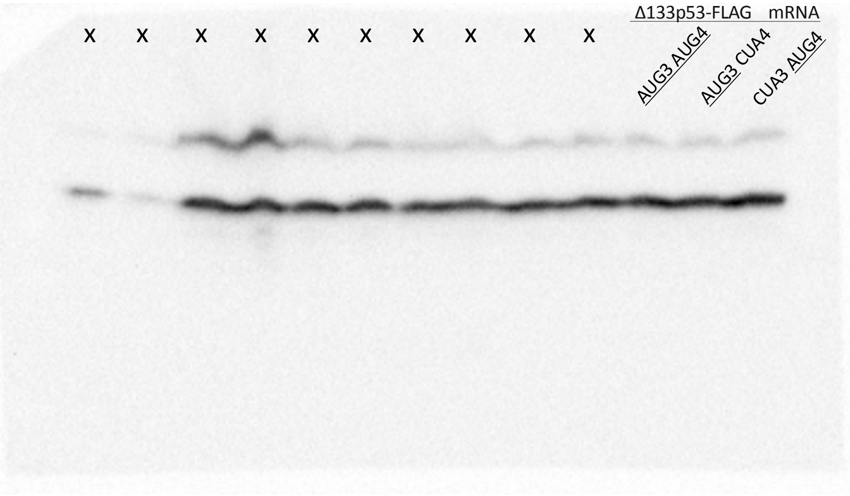

Figure 3D: method - radioactivity scanning

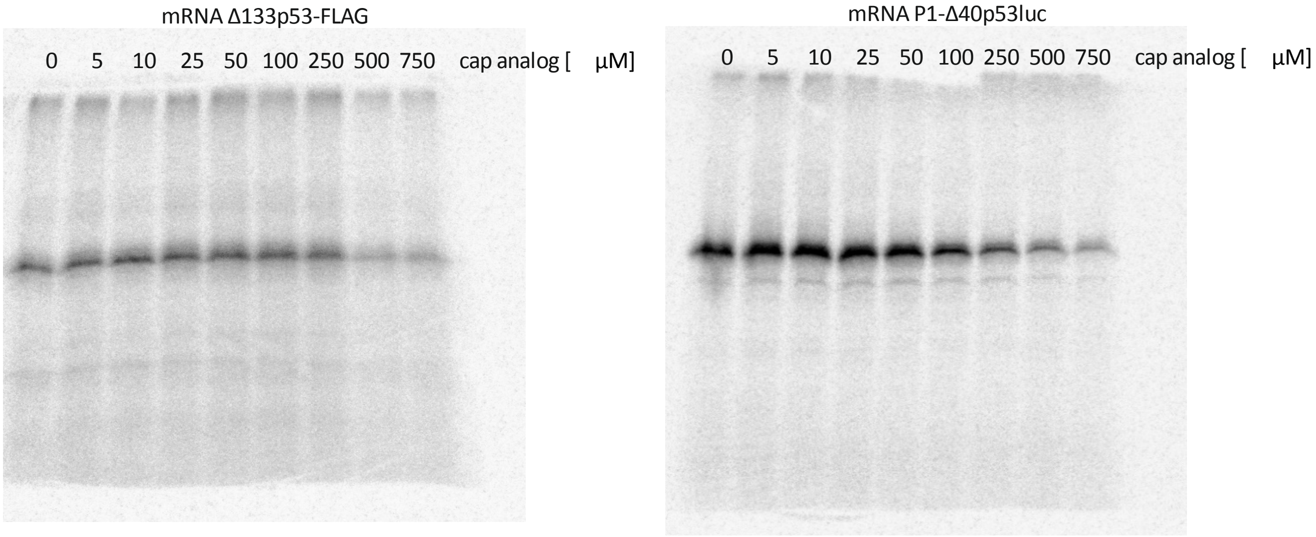

Figure 4: method – fluorescence digitalization

B:

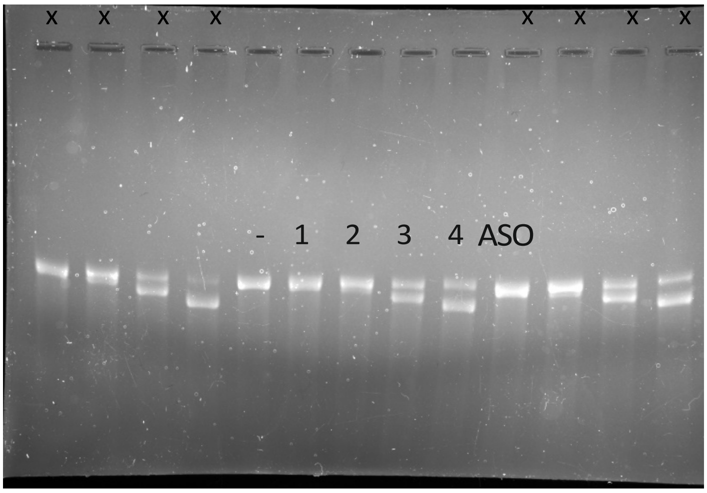

C:

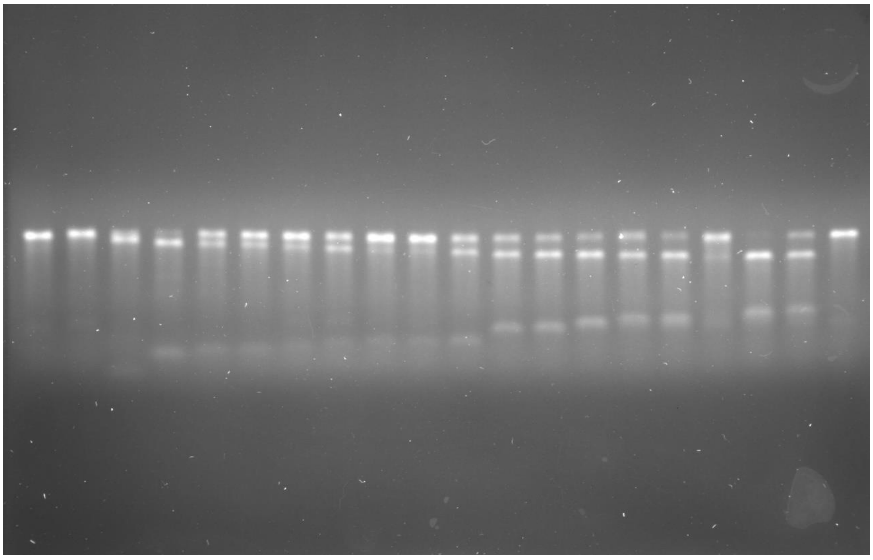

Figure 5: method - radioactivity scanning

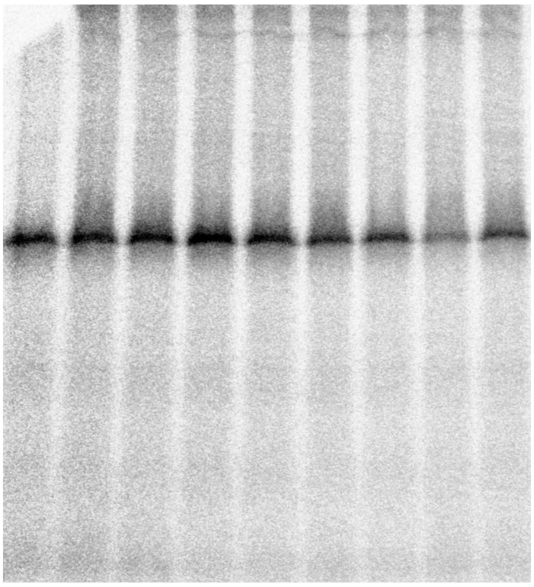

Fig. 6: method - chemiluminescence digitalization; membrane size

A: MCF-7

Δ133p53-FLAG

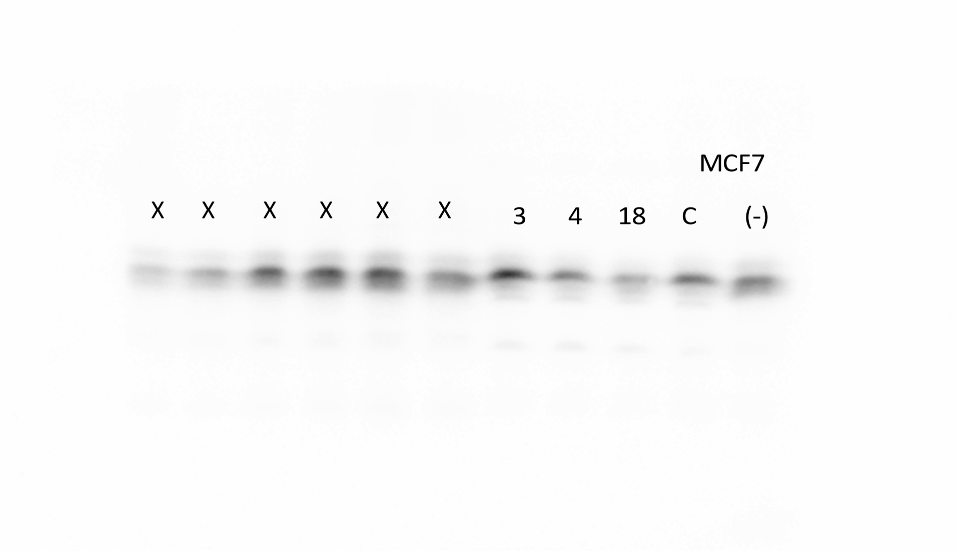

P53

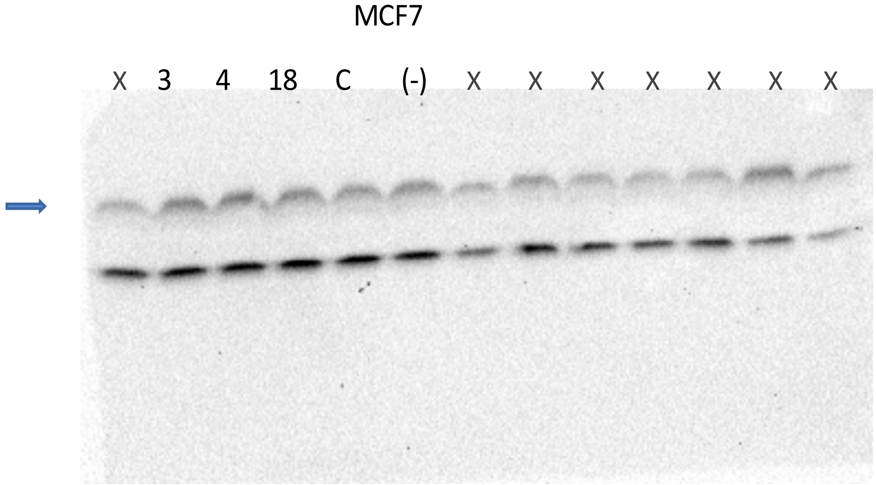

GAPDH

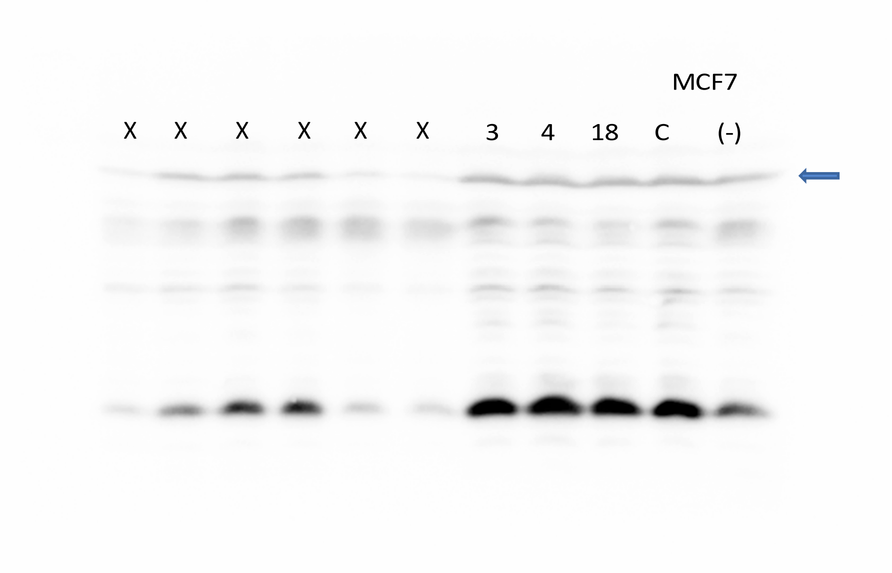

6B: HepG2

Δ133p53-FLAG

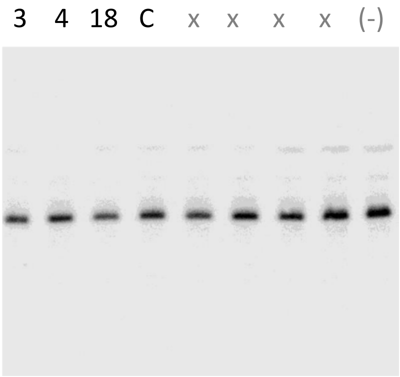

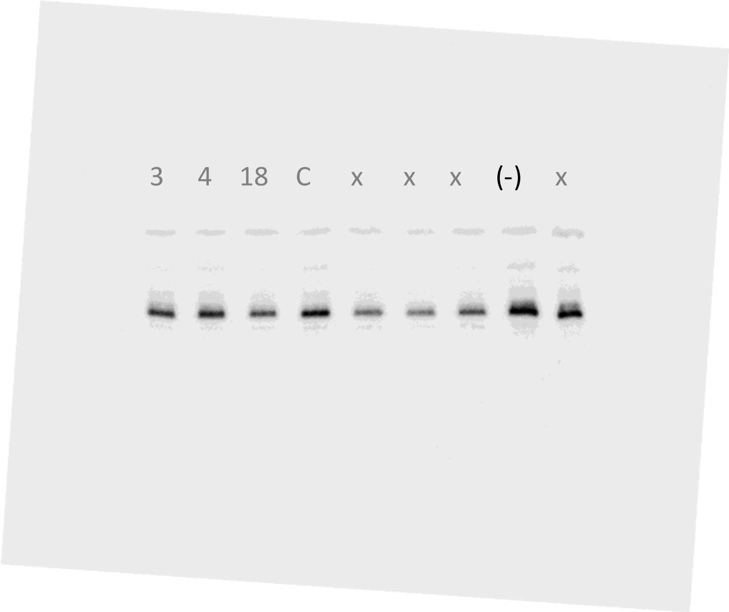

P53

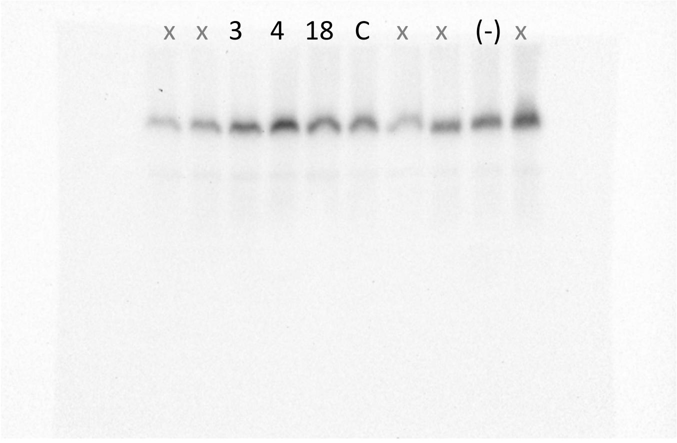

β-actin

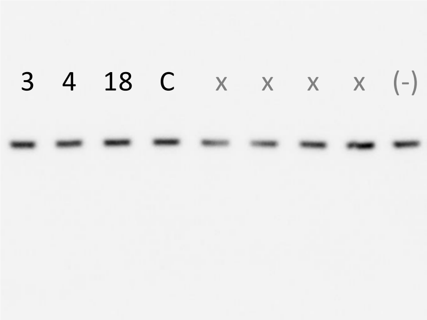

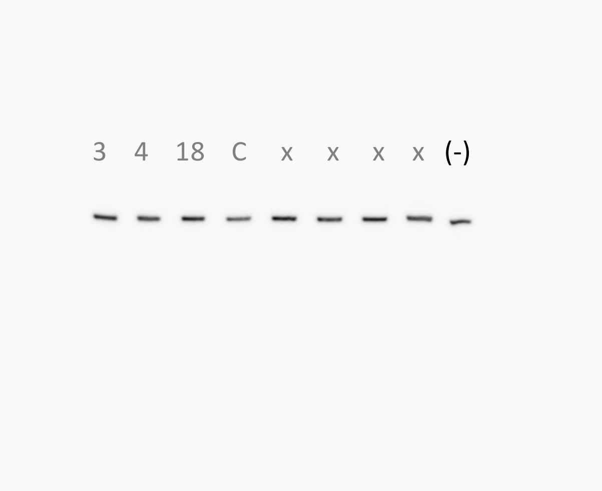

6 C: H1299

Δ133p53-FLAG

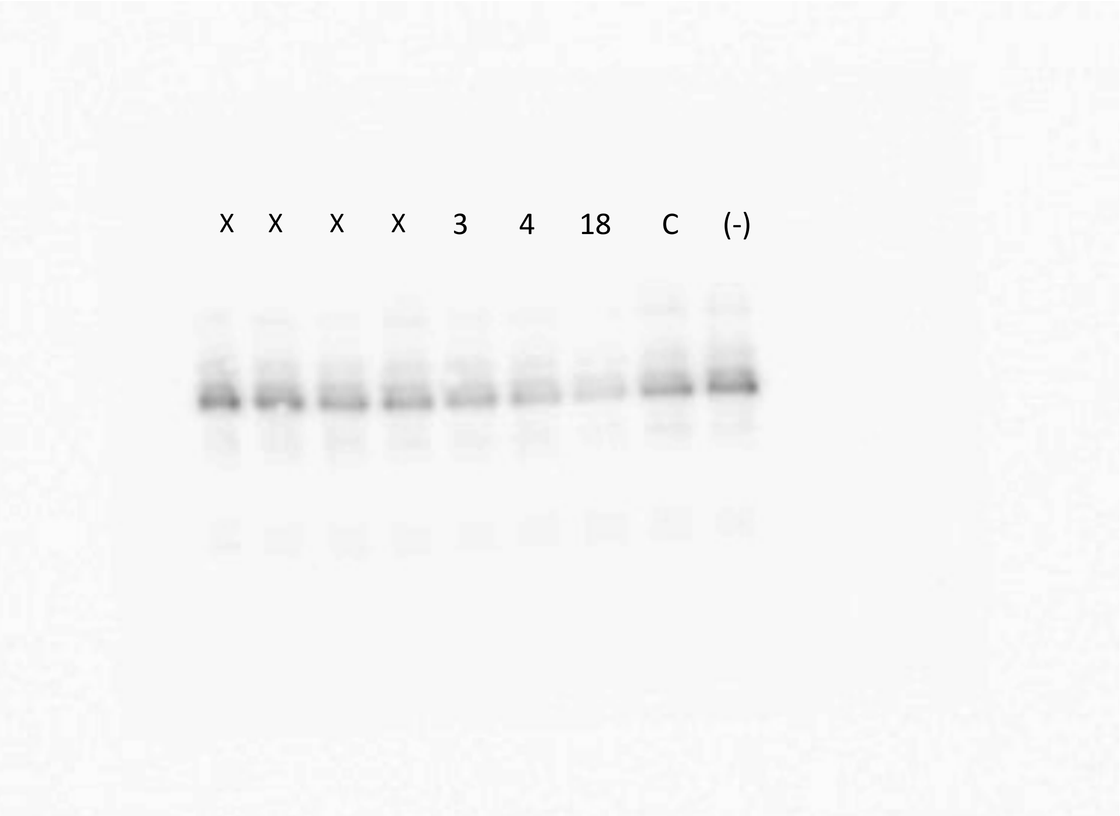

β-actin

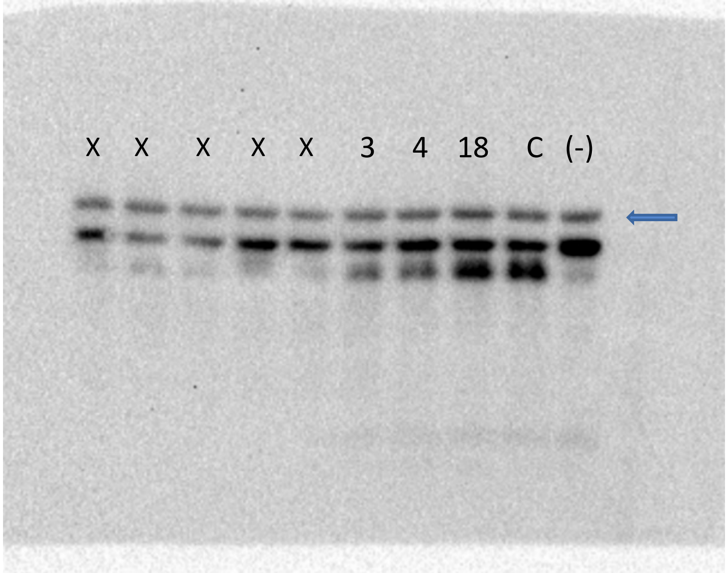

Fig. 7: method - chemiluminescence digitalization

A:

Δ133p53-FLAG

3,4,18 18,4 18,3 3,4 C3x C2x 3,C 4,C 18,C (-)

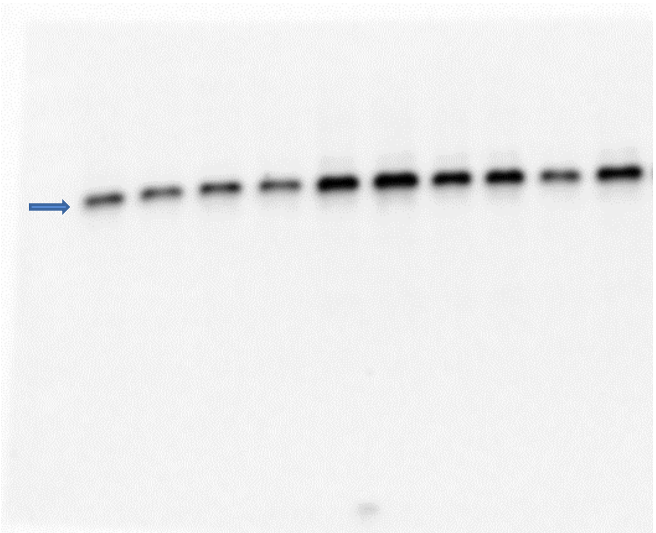

β-actin

3,4,18 18,4 18,3 3,4 C3x C2x 3,C 4,C 18,C (-) ASO

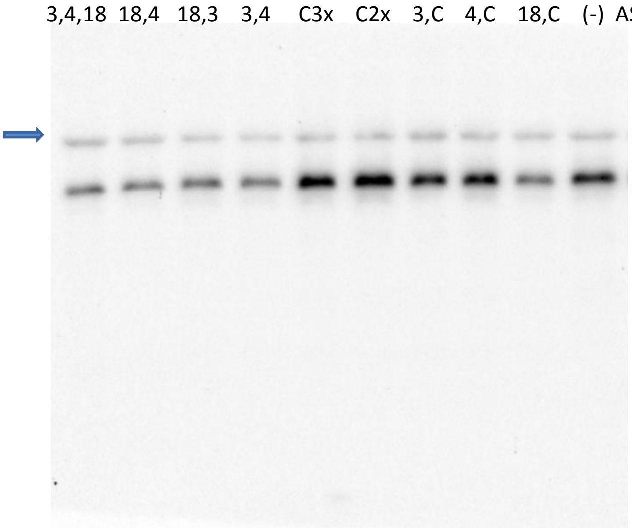

B:

Δ133p53-FLAG

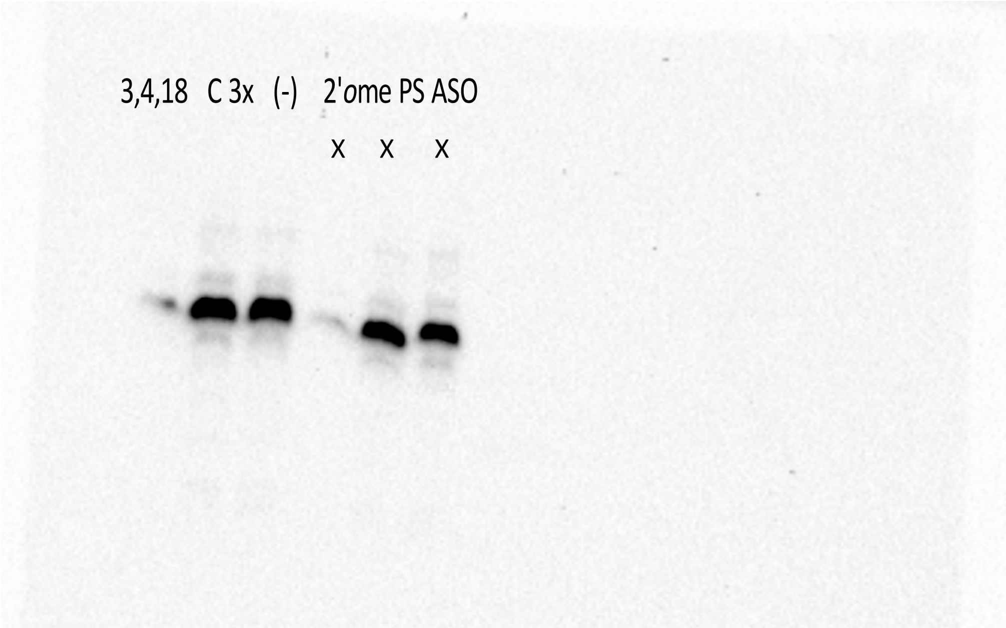

p53

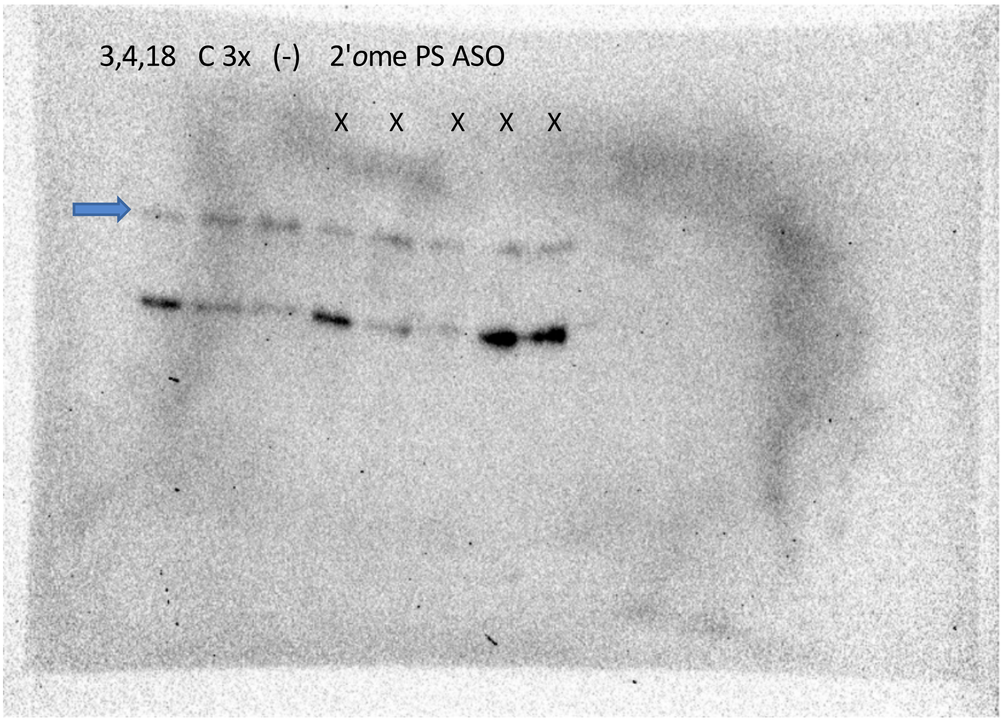

β-actin

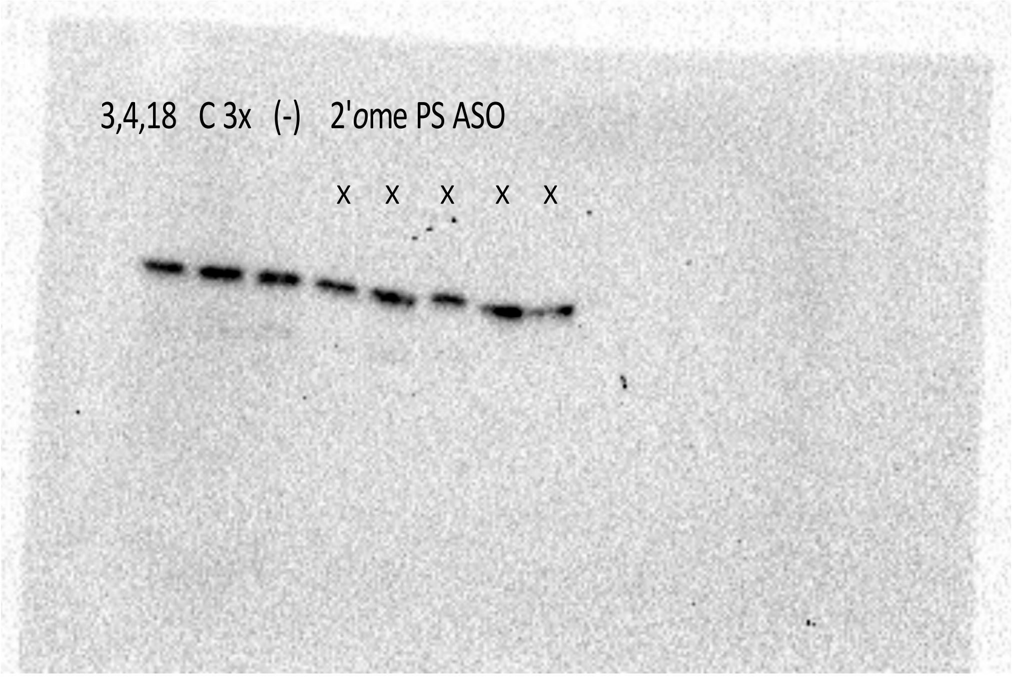

Supplement: S1 Raw images — (PDF) [file pone.0256938.s002.pdf]
